# Integrated phenotyping of the anti-cancer immune response in HIV-associated hepatocellular carcinoma

**DOI:** 10.1016/j.jhepr.2023.100741

**Published:** 2023-03-22

**Authors:** David J. Pinato, Takahiro Kaneko, Antonio D’Alessio, Alejandro Forner, Petros Fessas, Beatriz Minguez, Edoardo G. Giannini, Federica Grillo, Alba Díaz, Francesco A. Mauri, Claudia A.M. Fulgenzi, Alessia Dalla Pria, Robert D. Goldin, Giulia Pieri, Pierluigi Toniutto, Claudio Avellini, Maria Corina Plaz Torres, Ayse U. Akarca, Teresa Marafioti, Sherrie Bhoori, Jose María Miró, Mark Bower, Norbert Bräu, Vincenzo Mazzaferro

**Affiliations:** 1 Department of Surgery & Cancer, Imperial College London, Hammersmith Hospital, Du Cane Road, London, UK; 2 Division of Oncology, Department of Translational Medicine, University of Piemonte Orientale, Novara, Italy; 3 Tokyo Medical and Dental University, Tokyo, Japan; 4 Liver Unit, Barcelona Clinic Liver Cancer (BCLC) Group, ICMDM, Hospital Clinic Barcelona, IDIBAPS. University of Barcelona, Barcelona, Spain; 5 National Biomedical Research Institute on Liver and Gastrointestinal Diseases (CIBEREHD), Instituto de Salud Carlos III, Madrid, Spain; 6 Liver Unit, Department of Internal Medicine Hospital Universitari Vall d’Hebron, Universitat Autonoma de Barcelona, Barcelona, Spain; 7 Vall d’Hebron Institute of Research (VHIR), CIBERehd Vall d’Hebron, Barcelona Hospital Campus, Barcelona, Spain; 8 Gastroenterology Unit, Department of Internal Medicine, University of Genoa, IRCCS-Ospedale Policlinico San Martino, Genoa, Italy; 9 Pathology Unit, Department of Surgical Sciences and Integrated Diagnostics, University of Genoa, IRCCS-Ospedale Policlinico San Martino, Genoa, Italy; 10 Pathology Department, Hospital Clínic, University of Barcelona, Barcelona, Catalonia, Spain; 11 Medical Oncology Department, Fondazione Policlinico Universitario Campus Bio-Medico, Rome, Italy; 12 National Centre for HIV Malignancy, Department of Oncology, Chelsea & Westminster Hospital, London, UK; 13 Centre for Pathology, Imperial College London, London, UK; 14 Hepatology and Liver Transplantation Unit, Department of Medical Area (DAME), University of Udine, Udine, Italy; 15 Azienda Ospedaliero-Universitaria “Santa Maria della Misericordia”, Institute of Histopathology, Udine, Italy; 16 Department of Histopathology, University College London Hospital, London, UK; 17 Hepato-Pancreatic-Biliary Surgery and Liver Transplantation, Fondazione IRCCS Istituto Nazionale Tumori, Milan, Italy; 18 Department of Infectious Disease, Hospital Clinic-IDIBAPS, University of Barcelona, Barcelona, Spain; 19 CIBERINFEC, Instituto de Salud Carlos III, Madrid, Spain; 20 James J. Peters VA Medical Center, Bronx, New York, NY, USA; 21 Icahn School of Medicine at Mount Sinai, New York, NY, USA; 22 Department of Oncology, University of Milan, Milan, Italy

**Keywords:** HCC, HIV, PD-L1, Prognosis

## Abstract

**Background & Aims:**

HIV-seropositivity shortens survival in patients with hepatocellular carcinoma (HCC). Although risk factors for HCC including HCV infection can influence T cell phenotype, it is unknown whether HIV can influence functional characteristics of the T cell infiltrate.

**Methods:**

From the Liver Cancer in HIV biorepository, we derived 129 samples of transplanted (76%) or resected (20%) HCC in eight European and North American centres. We profiled intra- and peritumoural tissue to evaluate regulatory CD4+/FOXP3+ and immune-exhausted CD8+/PD1+ T cells in HIV+ (n = 66) and HIV- (n = 63) samples. We performed targeted transcriptomics and T-cell receptor sequencing in a restricted subset of samples evaluated in relationship with HIV status. We correlated immunopathologic features with patients’ characteristics including markers of HIV infection.

**Results:**

Of the 66 HIV+ patients, 83% were HCV coinfected with an undetectable HIV viral load (51%) and a median blood CD4+ cell count of 430 cells/mm^3^ (range 15–908). Patients who were HIV+ were compared with HIV- controls with similar staging characteristics including Barcelona Clinic Liver Cancer (BCLC) stage A–B (86% *vs.* 83%, *p* = 0.16), <3 nodules (90% *vs.* 83%, *p* = 0.3) and median alpha-foetoprotein values (10.9 *vs.* 12.8 ng/ml, *p* = 0.72). HIV+ samples had higher PD-L1 expression rates in tumour tissue (51% *vs.* 8% *p* <0.0001) and displayed denser intratumoural CD4+/FOXP3+ (*p* <0.0001), CD8+/PD1+ (*p* <0.0001), with lower total peritumoural CD4+ (*p* <0.0001) and higher peritumoural CD8+/PD1+ (*p* <0.0001). Gene set analysis revealed HIV+ cases to have evidence of dysregulated adaptive and innate immunity. Tumour-infiltrating lymphocyte clonality was not influenced by HIV status.

**Conclusions:**

HIV-associated HCC harbours a profoundly immune-exhausted tumour microenvironment, warranting prospective testing of immunotherapy in this treatment-deprived patient population.

**Impact and Implications:**

Hepatocellular carcinoma is a non-AIDS defining malignancy characterised by poor survival. The programmed cell death (PD-1) pathway governs antiviral and anticancer immune exhaustion and is a therapeutic target in HCC. This study highlights how HIV infection is associated with significantly higher PD-L1 expression in HCC cells and in the surrounding microenvironment, leading to changes in cytotoxic and regulatory T cell function and dysregulation of proinflammatory pathways. Taken together, our results suggest dysfunctional T cell immunity as a mechanism of worse outcome in these patients and suggest clinical testing of checkpoint inhibitors in HIV-associated HCC.

## Highlights

• HIV+ resected HCCs are characterised by an increased expression rate of PD-L1.
• HIV+ samples had an intratumoural infiltrate enriched with regulatory T cells and immune-exhausted CD8^+^PD-1^+^ T cells compared with HIV-HCC.
• HIV+ HCCs show a downregulation of gene signatures related to innate and adaptive immune response on targeted transcriptomic analyses.
• No association was found between HIV infection and T cell clonality within the intratumoural infiltrate.

## Graphical abstract

**Figure:**
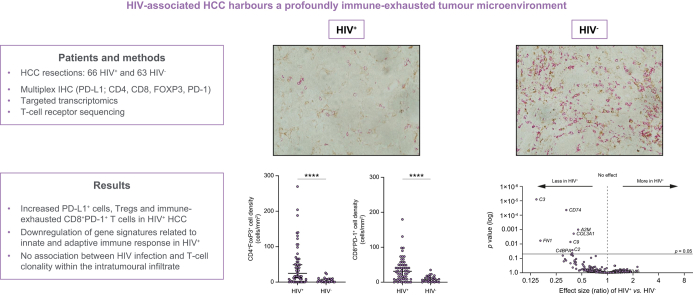

## Introduction

Hepatocellular carcinoma (HCC) is the sixth most common cancer worldwide and fourth major cause of cancer-related mortality, being responsible of more than 600,000 deaths annually.^1^

In people living with HIV (PLHIV), HCC has rapidly become one of the major determinants of morbidity and mortality, especially in patients who are coinfected with HBV or HCV,^2^ where HCC accounts for nearly half of liver-related mortality.^3^ In a previous multicentre study, we have shown that HIV infection increases the risk of death by 24% compared with HIV- controls despite adequate antiretroviral (ARV) therapy.^4^ Although evolving epidemiological data confirm a clinically important association between HIV infection and the prognosis of HCC, none of the studies published so far contribute to explaining whether the adverse course that characterises HIV-associated HCC is linked to intrinsically adverse biology rather than socio-economic disparities.^5^^,^^6^

Impairment of adaptive immunity, and T cell function in particular, is one of the key features of HIV infection that critically affects the pathogenesis of HCC. HIV coinfection synergises with hepatotropic viral infection largely through immune dysregulation, which promotes faster fibrosis^7^ and accelerated oncogenesis.^8^ Lower peripheral CD4 counts lead to a higher risk of HCC,^9^ tracing a link between HIV-induced immune dysfunction and cancer immune-surveillance.

T cell exhaustion is a major contributor to the pathogenesis of both HCC and HIV.^10^ Both conditions are characterised by persisting antigen presentation and inability to remove the pathogenic *noxa*,^11^ a state capable of shifting T cells to a dysfunctional phenotype characterised by the expression of high levels of co-inhibitory receptors such as cytotoxic T-lymphocyte antigen 4 (CTLA-4), the programmed cell death-1 receptor (PD-1), and its ligands (PD-L1/PD-L2), all of which lead to impaired effector cytokine production.^12^ Although necessary to prevent tissue damage from excessive immune reaction to chronic infection,^13^ T cell exhaustion is also key tumorigenic mechanism in HCC, gearing the liver microenvironment towards immunosuppression.^14^

The PD-1/PD-L1 pathway is central to both HIV-related T cell exhaustion^15^ and in the pathogenesis of HCC,^16^ where PD-L1 overexpression is common and predicts for adverse clinical course.^17^ PD-1/PD-L1 blocking antibodies have demonstrated antitumour activity in HCC and have become the backbone of immunotherapy combinations in association with anti-angiogenics and CTLA-4 antagonists.^14^ Although insufficient to demonstrate a significant survival benefit as a first and second-line systemic therapy for advanced HCC, blockade of the PD-1 pathway alone induces measurable responses in nearly 20% of patients.^18^^,^^19^

HIV-associated malignancies are excluded from clinical trials of immune checkpoint inhibitors (ICIs) because of concerns over safety and reduced efficacy. Evidence from observational and prospective clinical trials in lung cancer and Kaposi sarcoma revealed PD-1 monotherapy to be safe and effective in PLHIV.^20^ The immune contexture of HCC is however profoundly different compared with other oncological indications and more prominently geared towards intrinsic immunosuppression.^14^ Whether HIV infection is a determinant of T cell dysfunction within the HCC microenvironment is currently unknown. As ICIs are gaining momentum in the systemic management patients with HCC, understanding whether HIV might impact responsiveness to immunotherapy is of utmost importance not only to optimise drug development, but also to facilitate clinicians in the routine prescribing of ICIs in clinical practice.

In answer to these unmet needs, we designed this study to portray the functional characteristics of the T cell infiltrate of HIV-associated HCC and verify the overarching hypothesis that tumour-induced T cell dysfunction might be influenced by HIV status and mechanistically involved in determining the poorer prognosis of HIV-associated HCC compared with HIV-negative patients.

## Patients and methods

### Patients and specimen collection

The Liver Cancer in HIV is a multicentre, prospectively maintained database of patients diagnosed with HIV-associated HCC that capitalises on a global network of investigators from 44 referral centres providing specialist multidisciplinary care for HIV and HCC across nine countries.^4^^,^^21^ Clinical outcomes of this dataset have been previously published.^4^ A biorepository of patients’ archival samples was generated including cases with histological diagnosis of HCC based on international guidelines.^22^ At the censoring date of 1 October 2019, the repository included a total number of 63 patients with HIV-associated HCC and 66 HIV-negative controls diagnosed in eight tertiary referral centres for the care of HCC. Patient disposition across participating institutions is documented in Table S1.

Archival, formalin-fixed paraffin-embedded (FFPE) material from diagnostic biopsy (n = 20) or surgical specimens (n = 109) was retrieved and reviewed locally for accuracy of histological diagnosis and adequacy of tissue for subsequent analyses. Tissue quality control was performed centrally following review of newly cut H&E sections by two consultant histopathologists (RG and FAM).

Clinicopathological variables reflective of oncological features and liver functional reserve at diagnosis, therapy for HCC and HIV infection were recorded following medical notes review.

Ethical approval for the utilisation of the tissues and for the analysis of the data was granted by the Imperial College Tissue Bank (Reference: R16008). Because of the retrospective nature of the study and the anonymous nature of the data analysed, informed consent of the patients was not required.

### Immunohistochemistry

Immunohistochemistry (IHC) staining was performed at the Imperial College Histopathology Laboratory (Hammersmith Hospital, London, UK) using the Leica Bond RX stainer (Leica, Buffalo, IL, USA). Tissue sections (2 μm thick) underwent single marker immunostaining for PD-L1 using antibody clone E1L3N (Cell Signalling, MA, USA, Cat. Nr. 13684). Multiplex immunostaining for CD4 (Spring Biosciences, Pleasanton, CA, USA, clone SP35), CD8 (Spring Biosciences, clone SP239), FOXP3 (Biolegend, San Diego, CA, USA clone 259D), and PD-1 (Spring Biosciences, clone NAT 105/E3) was performed at University College London using a pre-optimised protocol.^23^

Evaluation of PD-L1 expression was performed in tumour cells and in tumour infiltrating T lymphocytes (TILs). We classified tumoural PD-L1 expression categorically using the tumour proportion score method (TPS), defined as percentage of viable tumour cells showing partial or complete membrane staining at any intensity. A TPS score of 1% was utilised to define PD-L1 positivity, in line with the cut-off routinely used in clinical trials of ICI.^24^

For multiplex IHC experiments, individual count of CD4+, CD8+, and CD4+/FOXP3+, CD8+/PD-1+ co-immunopositive cells was performed in tissue photomicrographs assessed at 40× magnification across tumoural and non-tumoural areas. Density of T cell infiltrate was calculated by manually computing the overall number of immunopositive cells per mm^2^ of tissue on the basis of the average of three independent readings as previously shown.^25^

### DNA/RNA purification

Following H&E-guided identification of target tumoural areas with >25% of viable tissue, RNA and DNA were purified from optimally de-paraffinised 10-μm-thick FFPE tissue sections for each sample using the Allprep DNA/RNA FFPE tissue kit (Qiagen, Venlo, The Netherlands, Cat. 80234). All the procedures followed the instructions of the manufacturer. RNA and DNA quantification and quality control were performed on an ND2000 Nanodrop spectrophotometer (Thermo Fisher Scientific, Loughborough, UK). DNA samples were further measured using a Qubit™ Flex Fluorometer 2.0 (Thermo Fisher Scientific).

### High-resolution T-cell receptor sequencing

We performed sequencing of the CDR3 variable regions of T-cell receptor-β (TCR-β) chains on purified DNA samples using the immunoSEQ Assay (Adaptive Biotechnologies, Seattle, WA, USA), as described previously.^26^ Clonality was computed on productive rearrangements and defined as 1-Peilou’s evenness.^27^ Normalisation of TCR-β template counts to total usable DNA was used to estimate T cell density. The quantity of usable DNA was determined by PCR amplification and sequencing of housekeeping genes expected to be present in all nucleated cells. Richness was calculated using the preseqR package.^28^ In total, 30 samples (15 HIV+, 15 HIV-) passed quality control criteria and were included in the final analysis.

### Nanostring immune profiling

We performed targeted transcriptomic profiling on total RNA samples derived from H&E-guided microdissection of target tumour tissue using the NanoString PanCancer Immune panel (Tables S2–S5) on an nCounter® Analysis System (NanoString Technologies, Seattle, WA, USA). Samples flagged for high normalisation values or with quality control standards falling outside default settings were examined carefully and a total of 48 samples (23 HIV+ and 25 HIV-) were included in the final analyses. We performed a gene set analysis (GSA) to investigate the differential regulation of 22 gene expression signatures on the basis of the HIV status.

### Statistical analysis

Patient characteristics were summarised as means or medians as appropriate, with Pearson’s Chi-Square or Fisher’s exact tests being utilised for the comparison of proportions between groups. We investigated correlations between clinicopathological variables using Pearson’s or Spearman’s correlation coefficient tests. Differences in medians across groups were evaluated using the Mann–Whitney *U* test. The Kaplan–Meier curve and log-rank test was used to evaluate differences in patients’ survival according to covariates of interest. In targeted RNA expression experiments, differential expression of genes of interest was determined using the false discovery rate method of Benjamini and Hochberg, with a predefined q-value of 5% as previously published.^29^

All statistical analyses were performed using SPSS version 26.0 (IBM Inc., Chicago, IL, USA) and GraphPad Prism v9.0 (GraphPad software Inc., La Jolla, CA, USA). All estimates were reported with 95% CIs and a two-tailed level of significance of *p* ≤0.05.

## Results

### Patient characteristics

Across HIV+ (n = 66) and HIV- patients (n = 63), the predominant aetiologic factor for chronic liver disease was HCV infection (83% and 59%, respectively). Clinical features of both groups including tumour stage, liver functional reserve, and therapy are presented in Table 1.

**Table 1:** **Comparison of clinical characteristics between HIV-negative and HIV-positive hepatocellular carcinoma (HCC) patients**.

Characteristic	HIV- (n = 63) (%)	HIV+ (n = 66) (%)
Age in years, median (range)	57 (41–71)	51 (41–64)
Sex (male/female)	55/8 (87/13)	57/9 (86/14)
Cirrhosis (present/absent)	63/0 (100/0)	62/4 (94/7)
Aetiology for HCC Hepatitis B Hepatitis C Alcohol excess Other	10 (16) 37 (59) 16 (25) 4 (6)	11 (17) 55 (83) 7 (11) 3 (5)
Barcelona Clinic Liver Cancer Stage 0/A B C D	45 (73) 12 (19) 1 (2) 4 (7)	50 (77) 6 (7) 9 (15) 1 (2)
Tumour node metastasis (TNM) stage I–II III–IV	59 (93) 4 (7)	57 (86) 9 (14)
Child–Turcotte–Pugh Class (A/B/C)	30/26/7 (47/42/11)	49/12/1 (79/19/2)
Alpha-foetoprotein (ng/ml), median (range)	12.8 (2–614)	9.0 (2–6,536)
Albumin (g/L), median (range)	3.6 (1.4–4.9)	4.0 (1.9–5.0)
Bilirubin (millimol/L), median (range)	29 (6–178)	18 (3–89)
Tumour size (cm), median (range)	2.0 (0.8–8.0)	2.5 (1.0–11.3)
Nodule (uninodular/multinodular)	15/46 (25/75)	36/30 (55/45)
Metastasis (present/absent)	4/59 (6/94)	9/57 (8/92)
HIV viral load (copies)∗, median (range)	—	0 (0–87,151)
Peripheral CD4 count (cells/mm^3^)† Median (range) <350 cells/mm^3^	—	430 (15–908) 19 (42%)
Interval from HIV diagnosis to HCC, median (range), years	—	19 (0–33)
Treatment for HCC Transplant (OLT) Resection Radiofrequency ablation (RFA) Trans-arterial chemoembolisation (TACE) Percutaneous ethanol injection (PEI) Hepatic arterial infusion (HAI) Trans-arterial radioembolisation (TARE) Systemic therapy	53 (84) 10 (16) 6 (10) 32 (51) 6 (10) 1 (2) 0 (0) 3 (5)	43 (65) 24 (36) 24 (36) 18 (27) 3 (5) 0 (0) 1 (2) 5 (8)
Overall survival (months), mean (95% CI)	113 (89–138)	111 (91–130)

∗ Available in 48 patients.

† Available in 45 patients.

Amongst HIV+ patients, most prevalent risk factor for HIV infection was history of intravenous drug abuse (n = 30, 45%). The mean duration from HIV infection to HCC diagnosis was 16 years (standard deviation, SD 10.7 years). Record on ARV therapy at the time of tissue sampling could be reconstructed in 59 patients (90%), 57 of whom were on ARVs at the time of HCC diagnosis. Most frequently used ARV classes were nucleoside reverse transcriptase inhibitors (n = 47, 82%) followed by non-nucleoside reverse transcriptase inhibitors (n = 22, 38%), integrase inhibitors (n = 14, 24%), and protease inhibitors (n = 12, 21%).

The majority of HIV+ patients were of Barcelona Clinic Liver Cancer (BCLC) stage 0/A (n = 48, 77%) and Child–Turcotte–Pugh (CTP) A class (n = 49, 79%).

At HCC diagnosis, 48 patients had an HIV RNA quantification available, 34 of them (71%) displaying evidence of an undetectable HIV viral load. Median blood peripheral CD4 counts (available in 45 patients) was 428 cells/mm^3^ (range 15–908). In terms of HCC therapy, patients most commonly received liver transplantation (n = 33, 71%) or resection (21, 22%).

Overall, the cohort of patients who were HIV- was selected as a control group (Table 1) and was balanced for key clinicopathologic features of HCC compared with patients who were HIV+, including sex (87% *vs.* 86% of males across groups, *p* = 0.88), presence of cirrhosis (100% *vs.* 94%, *p* = 0.07), proportion of patients in BCLC stage 0–A/B (92% *vs.* 84%, *p* = 0.27), alpha-fetoprotein (AFP) ≥400 ng/ml (4% *vs.* 9%, *p* = 0.65). The proportion of patients in CTP class A was significantly inferior in patients who were HIV- compared with those who were HIV+ (47% *vs.* 79%, *p* = 0.003). The overall survival (OS) in the two patient cohorts was not significantly different (Fig. S1, Log rank *p* = 0.49). Patients who were HIV- had a mean OS of 113 months (95% CI 88–138 months) and a median OS of 120 months (95% CI 74.9–166 months). For patients who were HIV+ the mean OS was 110 months (95% CI 91–130 months), whereas the median OS was not reached.

### HIV-associated HCC is characterised by distinctive phenotypic characteristics of the T cell infiltrate

**Fig. 1:**
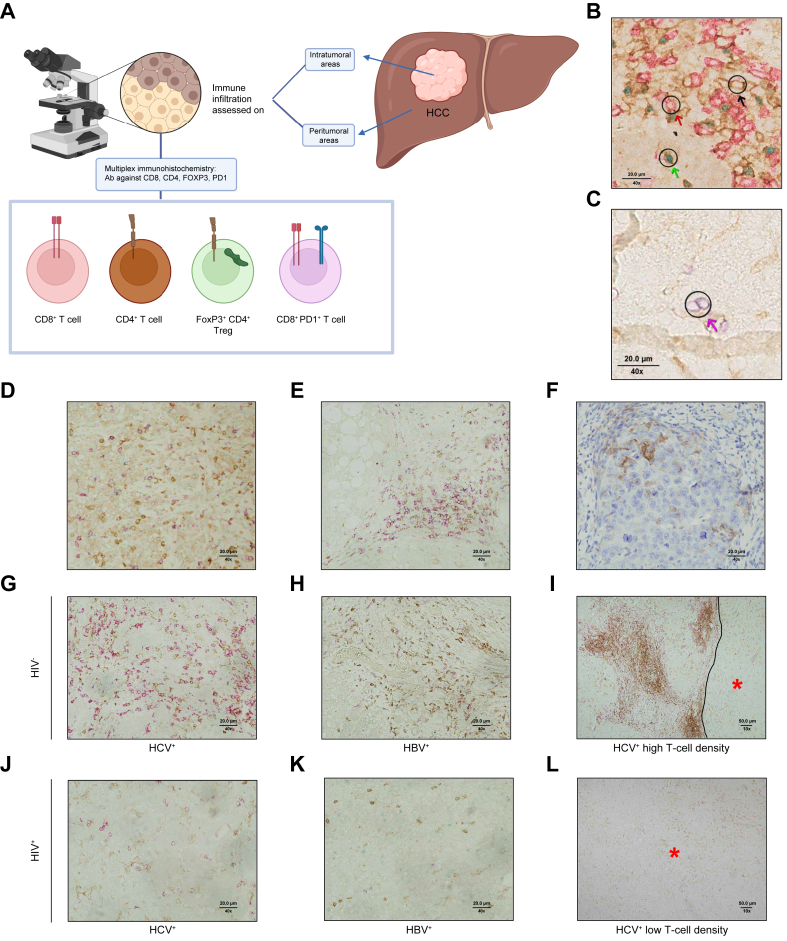
Multiplex immunohistochemistry (A) was used to assess phenotypic characteristics of the immune cell infiltrate as shown in representative sections. Sections were co-immunostained with four chromogens and assessed at 40× magnification to identify: CD8+ T cells (red arrow), CD4+ T cells (black arrow), CD4+/FOXP3+ T cells (green arrow) as shown in (B) and CD8+/PD1+ T cells (purple arrow, C). Representative sections showing evidence of T cell infiltration on the tumour tissue (D) and peritumoural liver tissue (E) as shown in a representative section of HIV-associated HCC. (F) Evidence of PD-L1 tumoural immunostaining in the same case depicted in D & E. (G and H) Representative tumoural sections of two HIV-associated HCCs arisen on the background of HCV (G) and HBV infection (H) both show evidence of a dense CD8/PD-1 (purple) and CD4/FOXP3 (brown/green). (J–L) Representative of matching HIV-negative HCV-positive (J) and HIV-negative HBV-positive cases (K) showing evidence of poor intratumoural T cell infiltration. Original magnification of (B–H) is 400×. (I and L) Representative sections of peritumoural tissue of an HIV-associated HCC case displaying evidence of high T cell infiltration (I) compared with a patient’s sample of HIV-negative HCC displaying evidence of poor peritumoural infiltration (L). An asterisk (∗) marks tumoural areas in both cases. Patient samples depicted in (I–L) were both derived from HCV-positive patients. Original magnification of (I–L) was 200×. Ab, antibody; HCC, hepatocellular carcinoma; PD-1, programmed cell death-1 receptor; Treg, regulatory T cell.

**Fig. 2:**
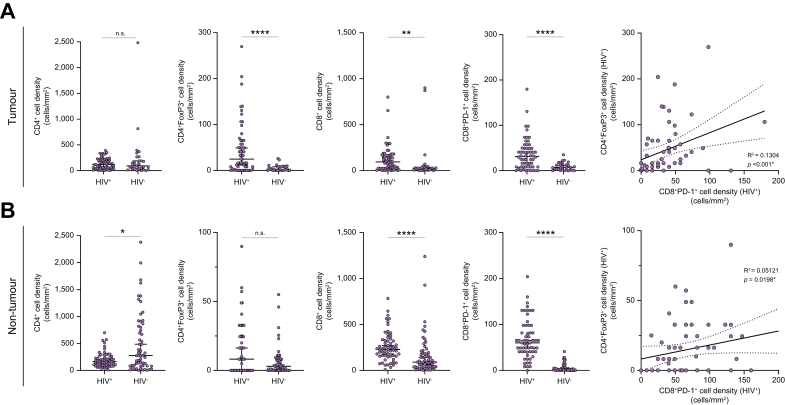
Scatter plots illustrating the distribution of immune infiltrating cells densities in the intratumoural (A) and peritumoural (B) areas assessed with multiplex immunohistochemistry. When compared to HIV-negative controls, HIV-positive samples showed a significant increase of intratumoural CD4+FOXP3+ cells (*p* <0.0001), total CD8+ cells (*p* = 0.0015), and CD8+PD-1+ cells (*p* <0.0001), with no difference in total CD4+ (*p* = 0.96) (A). In the peritumoural areas (B), CD4+ were higher in HIV-negative controls (*p* = 0.037) with no difference in CD4+/FOXP3+ (*p* = 0.10), whereas total CD8+ cells and CD8+/PD-L1+ cells were higher in HIV-positive samples (*p* <0.0001 for both associations). In intratumoural and peritumoural areas, a positive correlation was observed between CD8+PD-1+ and CD4+FOXP3+ cells. TILs distribution was compared across HIV-positive and negative with the Mann–Whitney *U* test. Correlation was assessed with Spearman’s correlation coefficient test. Statistical significance is reported as ∗*p* <0.05, ∗∗*p* <0.01, ∗∗∗∗*p* <0.0001. n.s., non-significant; PD-1, programmed cell death-1 receptor; TIL, tumour infiltrating T lymphocyte.

Each sample was evaluated for phenotypic characteristics of the T cell infiltrate by multiplex IHC on intratumoural (IT) and peritumoural (PT) areas. Representative sections are shown in Fig. 1. The overall distribution of CD8+, CD4+/FOXP3+, and CD8+/PD-1+ T cells across IT and PT areas is reported in Table S6. As shown in Fig. 2, IT areas of HIV+ samples had significantly higher median CD4+/FOXP3+ cells (2.5 *vs.* 24.7 cells/mm^2^, *p* <0.0001), total CD8+ (93.9 *vs.* 26 cells/mm^2^, *p* = 0.015) and CD8+/PD-1+ co-immunopositive cells (31.3 *vs.* 7 cells/mm^2^, *p* <0.0001) compared with HIV- samples. In PT areas the distribution of CD4+ (163.4 *vs.* 275.5 cells/mm^2^ *p* = 0.037) was lower in patients who were HIV+, whereas cell density of CD8+ (228.7 *vs.* 90.7, *p* <0.0001) and CD8+/PD-1+ T cells (60 *vs.* 2 cells/mm^2^, *p* <0.0001) but not CD4+/FOXP3+ T cells (8.2 *vs.* 3 cells/mm^2^, *p* = 0.1) was higher in individuals who were HIV+ compared with the HIV- counterparts. In patients who were HIV+, we found a positive linear correlation between CD8+/PD-1+ and CD4+/FOXP3+ T cells in IT (0.51, *p* <0.0001) and PT (Spearman R 0.30, *p* = 0.01) areas.

The distribution of the assayed T cell subgroups did not significantly differ based on CTP class scores neither in IT nor in PT areas (Table S7).

### Relationship between PD-L1 expression and phenotypic characteristics of T lymphocyte infiltrate

**Fig. 3:**
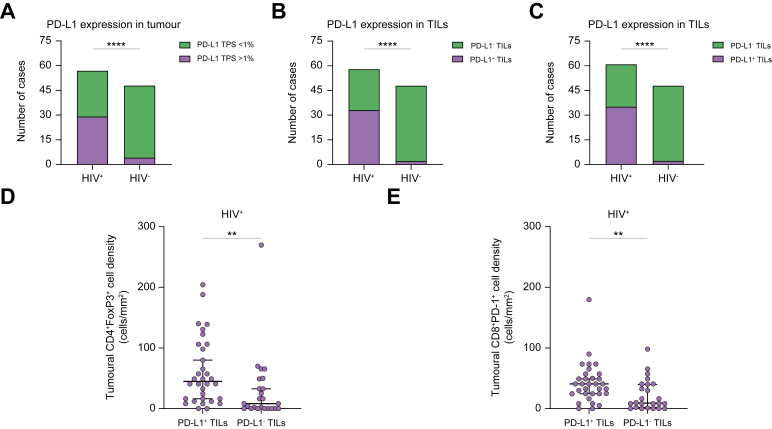
Histograms illustrating proportion of PD-L1 positivity across HIV-positive and negative samples in tumour areas (A) and in TILs distributed within tumoural areas (B) and in the peritumoural tissue (C). (D and E) The positive relationship between CD4+FOXP3+ and CD8+PD1+ cell density in samples categorised as positive or negative for PD-L1 expression in TILs of HIV+ cases. Differences across categories were tested with the Mann–Whitney *U* test. Statistical significance is reported as ∗. ∗*p* <0.05, ∗∗*p* <0.01, ∗∗∗∗ *p* <0.0001. The *p* values for associations are: (A, B, C), *p* <0.0001; (D), *p* = 0.0026; (E), *p* = 0.0072. PD-1, programmed cell death-1 receptor; PD-L1, programmed cell death receptor ligand 1; TILs, tumour infiltrating T lymphocytes.

PD-L1 status could be reconstructed in 105 cases (48 HIV- and 57 HIV+), owing to lack of sufficient material in 24 cases. Tumoural expression of PD-L1 as evaluated by a TPS ≥1% was fivefold higher in HIV+ 29/57 (51%) compared with HIV- samples 4/48 (8%, Fisher’s exact test *p* <0.0001 Fig. 3A), and higher compared with historical HIV- controls from the literature, where rates of PD-L1 tumoural immunopositivity are reported to be 17%.^17^ PD-L1 expression in the intratumoural immune cell infiltrate was significantly higher in HIV-associated HCC, 33/57 (58%) HIV+ specimens compared with 2/48 (4%) of HIV- controls (X^2^ *p* <0.0001, Fig. 3B). Similarly, we found higher prevalence of PD-L1-positive immune cell infiltrates in the background non-tumoural areas of HIV+ cases (35/57, 61%) compared with HIV- cases (2/48, 4%, X^2^ *p* <0.0001 Fig. 3C). PD-L1 immunopositive lymphocytes in tumour and non-tumour areas were not differentially distributed according to HBV (*p* = 0.86 and *p* = 0.22, respectively) nor HCV-related aetiology of chronic liver disease (*p* = 0.68, *p* = 0.38) in HIV-associated HCC patient samples.

Tumoural PD-L1 expression was associated with a denser intratumoural regulatory T cell infiltrated as evidenced by higher CD4+/FOXP3+ cell density (40.8 *vs.* 12.3 cells/mm^2^, *p* = 0.001, Fig. S2). Tumours harbouring PD-L1^+^ TILs were also the ones displaying higher CD4^+^/FOXP3^+^ (49.0 *vs.* 8.2 cells/mm^2^, *p* = 0.002) and CD8+/PD-1+ TIL cell density (40.8 *vs.* 12.3 cells/mm^2^, *p* = 0.016, Fig. 3D and E).

Tumoural PD-L1 expression was independent of key clinicopathologic features of HCC including BCLC stage (*p* = 1.0), CTP class (*p* = 0.403), AFP >400 ng/ml (*p* = 0.595), and presence of portal vein thrombosis (*p* = 1.0). A PD-L1 TPS score ≥1 was not associated with patient OS in HIV-associated HCC (log-rank test *p* = 0.41, Fig. S3).

We further investigated the relationship between characteristics of the tumour microenvironment of HIV-associated HCC and biomarkers of HIV infection. Peripheral CD4+ count was not associated with PD-L1 TPS scores. We found a weak positive correlation between peripheral CD4+ and peritumoural total CD4+ cells (R^2^=0.09, *p* = 0.044) but no correlation with either intra- or peritumoural CD4+/FOXP3+ and CD8+/PD-1+ TIL density. HIV viral load was similarly unrelated with PD-L1 TPS and phenotypic characteristics of the intra- and peritumoural T cell infiltrate (Fig. S4).

### HIV infection is associated with distinctive phenotypic features of T cell infiltrate but not clonality

**Fig. 4:**
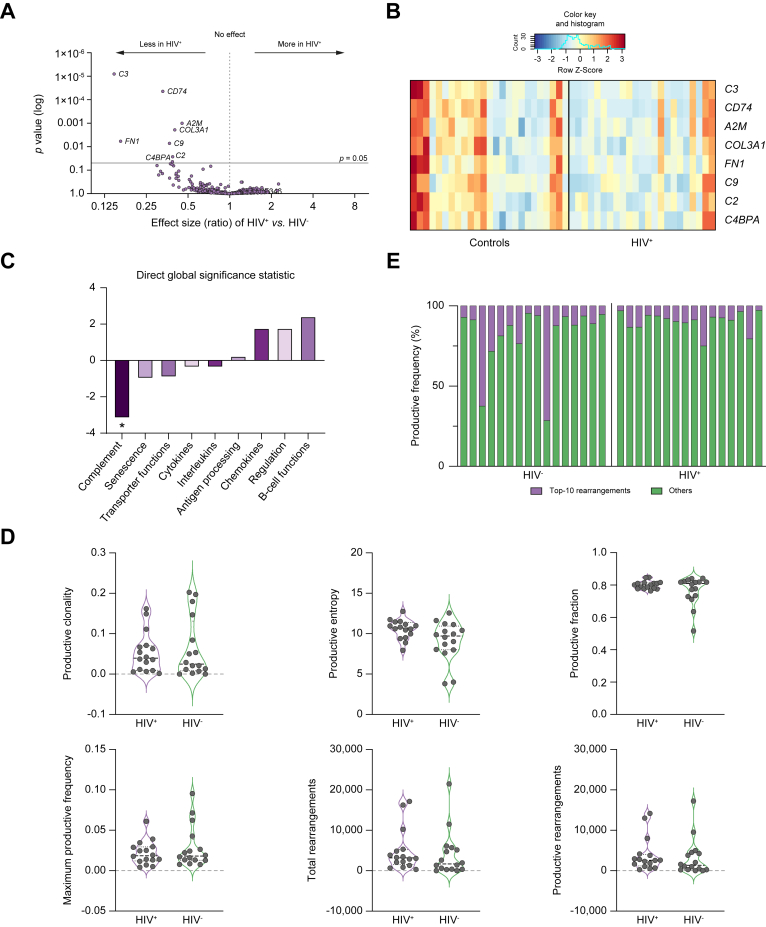
Volcano plot of differentially regulated genes identified by Nanostring analysis (A). The Benjamini–Hockberg *p* values are correlated to fold-changes in transcripts identified in HIV-positive HCC (n = 23) *vs.* HIV-negative controls (n = 25). Transcripts achieving statistical significance (*p* <0.05) are highlighted by the presence of the corresponding gene name. (B) Heatmap of the eight transcripts that are differentially regulated in HIV-associated HCC compared with controls. The z-score plotted in each cell represents the relative expression of the individual gene in each sample, resulting from the raw gene count minus the mean divided by the standard deviation of the gene distribution. (C) Graphical representation of the of the directed Global Significance Score of gene signatures across HIV-positive HCC and controls, with the complement-related signature reaching statistical significance, reported as ∗ (*p* <0.05). (D) Grouped violin plots illustrating different readouts of T cell clonality in the intratumoural infiltrate of HIV-positive samples *vs.* HIV-negative controls (n = 16 in each group). (E) Graphical illustration of the distribution of the top 10 T-cell receptor rearrangements as measured by productive frequency in HIV-positive *vs.* HIV-negative samples.

To complement the multiplex IHC experiments showing enrichment of CD4+/FOXP3+ and CD8+/PD-1 T cells in HCC samples of patients affected by HIV, we performed an exploratory targeted transcriptomic analysis of a smaller subset of 48 patient samples with viable tumour tissue (Table S8). We utilised the nCounter PanCancer Immune Profiling panel, accounting for 770 genes as detailed in Table S2, and we analysed 23 HIV+ and 25 HIV- samples to provide mechanistic insight into the molecular drivers characterising the tumour immune microenvironment in association with HIV infection (Fig. S5). By performing directed GSA, we demonstrated that compared to HIV- controls, patients with HIV-associated HCC demonstrated evidence of profound differences in terms of transcripts regulation. In particular, alongside a modest transcriptional repression of interleukins and cytokines, the functional domain of complement activity was particularly downregulated. We subsequently assessed differential expression of individual genes across HIV+/HIV- groups and demonstrated significant downregulation of a number of transcripts, including *C4B*, *C2*, *C3*, and *C9* in HIV-associated HCC, which code for factors of the complement cascade and is associated with inflammation and opsonisation of target cells (Fig. 4A–C).

Lastly, we hypothesised whether HIV infection might be associated with differences in T cell clonality and performed high-resolution TCR-β chain sequencing using the ImmunoSEQ assay in a subgroup with comparable staging and baseline clinico-pathologic characteristics (Table S8). As shown in Fig. 4D and E, we found no evidence for an association between HIV infection and T cell clonality within the intratumoural infiltrate as measured by a number of reproducible readouts. We assessed productive clonality, a normalised score based on diversity and sample entropy where higher values represent samples with fewer predominant rearrangements, productive entropy (frequencies of all productive sequences divided by the logarithm of the total number of unique productive sequences), and distribution of the 10 most frequently identified clonotypes across sample groups.

## Discussion

The PD-1 pathway plays a crucial role in the induction and persistence of T cell tolerance against cancer and viral *noxae*.^30^ Inhibition of the PD-1/PD-L1 interaction is the backbone of several therapeutic combinations that are revolutionising standards of care in advanced HCC. Although ICIs appear to be safe in PLHIV, whether the remarkable advances offered by immunotherapy in HCC can be extended to PLHIV remains largely unknown.^31^

Taking advantage of a large, multicentre repository of patient samples collected as part of the Liver Cancer in HIV registry, we performed multitechnology assessment of T cell phenotype and function in archival HCC samples of patients with and without HIV.

Despite evidence of well-controlled HIV infection as evidenced by undetectable HIV RNA and preserved peripheral blood CD4+ cell counts in the vast majority of patients, we found that the tumour microenvironment of HIV-associated HCC patients was characterised by stronger tumoural PD-L1 expression and denser intratumoural CD8+/PD-1+ and CD4+/FOXP3+ cell infiltration, suggesting evidence of more profound T cell dysfunction in HIV+ cases compared with controls. Regulatory T cells are frequently recruited as immune-suppressive cells within the HCC microenvironment^32^ and higher expression of regulatory T cell transcripts is associated with poorer prognosis in this tumour.^33^ Similarly, the presence of immune-exhausted CD8+/PD-1+ T cells is highly indicative of a defective cytotoxic capacity, leading to unopposed malignant disease progression. Recently, CD8+/PD-1+ T cells have been implicated in the reduced sensitivity to PD-1 inhibition in animal models of HCC secondary to non-viral aetiology, further highlighting the adverse role of these T cell subset in driving disease progression and response to therapy.^34^

Although multiplex immunohistochemistry revealed a highly significant difference in the distribution of immune-exhausted CD8 T cells and regulatory CD4 T cells depicting a higher degree of T cell dysfunction in HIV+ cases, transcriptomic experiments complement these findings by emphasising a greater role in the differential regulation of pro-inflammatory pathways including evidence of complement downregulation, alongside dysregulation of cytokine and chemokine pathways.

In particular, we found that HIV+ samples had a significant reduction of transcripts linked to innate immune response, such as A2M and FN1, both coding for acute phase proteins, and of a key mediator of the adaptive response such as CD74, which is involved in antigen recognition and CD4+ T cell function.

The most profound downregulation was found in transcripts related to the complement cascade (C2, C3, C4BPA, C9). Complement plays a complex and often dual tumour promoting and opposing role in cancer, with C3 and C4b, the factors emerging as more strongly dysregulated in HIV+ cases being intimately linked to the promotion of angiogenesis.^35^ In cancers including HCC, release of complement mediators such as C2 and C3 has been linked to macrophage polarisation and TIL functional reprogramming, raising questions as to their potential role as a therapeutic target for cancer immunotherapy.^36^

Although complement has been traditionally linked to opsonisation and subsequent innate immune activation following injury, a growing body of evidence has shown a non-canonical regulatory role of the complement system, with effector T cells upregulating complement gene transcription as an intrinsic cellular mechanism of metabolic regulation.^37^

Despite significant differences in T cell phenotype observed by immunohistochemistry, high-resolution TCR-β chain sequencing showed comparable distribution of multiple readouts of T cell clonality in HIV+ *vs.* HIV- samples. Clonality of T cell response as measured by the richness in V-D-J receptor sequences identified within tumour samples has been highlighted as one of the characteristics associated with stronger probability of effective anticancer immune reconstitution following T cell immune checkpoint blockade,^38^^,^^39^ whereas an increased post-immunotherapy peripheral clonality has been related to improved long-term outcomes in other cancer types.^40^ In HCC, translational and clinical data suggest that reversal of T cell exhaustion through blockade of the PD-1/PD-L1 pathway, although effective, requires combined modulation of multiple co-inhibitory pathways that affect myeloid and other stromal components of the tumour microenvironment,^41^ suggesting therefore that T cell clonality may not be univocally associated with immune-responsiveness unlike melanoma and other more immune-sensitive malignancies.^42^

Based on our data, HIV infection does not appear to influence T cell clonality, suggesting that other factors such as underlying hepatotropic viral infection status or differential enrichment in tumour-associated antigens to be potentially contributory to T-cell receptor diversity. The lack of complimentary TCR-α chain sequencing data precludes us from drawing definitive conclusions as to the antigen-dependence of T cell infiltration: a point that should be clarified in follow-on studies.

To our knowledge, despite being limited by its retrospective design, this is the largest study to describe the immune phenotype of HIV-associated HCC. Because none of the patients in this study were treated with ICIs, we cannot draw conclusions as to whether the characteristics of profound immune-suppression seen in HIV-associated cases might be predictive of response to immunotherapy. If a simplistic and pragmatic classification strategy were to be followed, the abundance of TILs and evidence of concomitant increased PD-L1 expression in HIV-associated HCC, portrays these tumours as bearing a ‘Type-I’ microenvironment, where a T cell reaction against cancer exists but is downregulated by cancer-driven immune-tolerogenic signals.^43^ However, the predictive power of microenvironment phenotyping based on TIL and PD-L1 expression, has demonstrated weaker predictive potential outside melanoma and non-small cell lung cancer, where, unlike HCC, PD-L1 has emerged as a companion diagnostic tool for the identification of patients who may benefit from immunotherapy.^44^ Interestingly, evidence of upregulation of pro-inflammatory pathways has been reported as a feature of spontaneous immunogenicity in HCC, a trait portending to favourable responsiveness to combination immunotherapy.^45^

Although evidence of an ‘immune subclass’ exists on the basis of broad RNA-sequencing profiles, methodological differences between our study and bulk RNA-seq datasets do not allow a direct phenotypic comparison across studies.^46^

Taken together, our study provides for the first-time evidence of stronger T cell dysfunction in patients with HIV-associated HCC. This finding is in keeping with the T cell impairment induced by HIV chronic infection,^10^ and it might explain the worse prognosis that we observed for HIV+ patients compared with HIV- controls.^4^ Phenotypic characteristics of the T cell infiltrate were not associated with severity of HIV infection in our study, although it should be noted that the majority of patients were on suppressive ARV. Although limited by its retrospective nature and by the choice of consecutive patients who had tissue available for analysis, our study is naturally skewed towards early-stage HCC patients, leaving open questions around the molecular characteristics of more advanced patients, who are usually diagnosed based on radiologic criteria. Another aspect to consider is the reliance of our study on archival material, an approach that prevents more in-depth functional studies of the tumour microenvironment and precludes antigen discovery experiments.^47^ The use of bulk RNA transcriptomics limits the capacity to detect small variations in the abundance of transcripts characterised by focal expression in restricted cell types, which could partly explain why some biomarkers (namely FOXP3 and PD-L1) were found to be differently expressed in the IHC experiments but not in the targeted transcriptomic analyses: a limitation that can only be overcome by single-cell RNA sequencing approaches using fresh tissue. This study is however strengthened by its international, multi-institutional accrual, capturing patients from diverse geographical origins, and by matching for basic clinicopathologic characteristics including stage and type of therapy.

Prospective clinical trials should investigate safety and efficacy of ICI therapy in HIV-associated HCC.

## Financial support

DJP is supported by grant funding from the 10.13039/100010269Wellcome Trust Strategic Fund (PS3416), the ASCO/10.13039/100000982Conquer Cancer Foundation Global Oncology Young Investigator Award 2019 (14704), 10.13039/501100000289Cancer Research UK (C57701/A26137), CW+ and the 10.13039/100011333Westminster Medical School Research Trust (JRC SG 009 2018-19), the 10.13039/501100023178Cancer Treatment and Research Trust (CTRT), and from the 10.13039/501100005010Associazione Italiana per la Ricerca sul Cancro (AIRC MFAG Grant ID 25697). DJP acknowledges infrastructural and grant support from the NIHR 10.13039/100016338Imperial Experimental Cancer Medicine Centre and the Imperial College BRC. AD is supported by the 10.13039/501100013342NIHR Imperial BRC and by grant funding from the 10.13039/501100009253European Association for the Study of the Liver (Andrew Burroughs Fellowship) and from Cancer Research UK (RCCPDB-Nov21/100008). AF is supported by a grant from 10.13039/501100004587Instituto de Salud Carlos III (PI18/00542). JMM received a personal 80:20 research grant from Institut d’Investigacions Biomèdiques August Pi i Sunyer (IDIBAPS), Barcelona, Spain, during 2017–23. BM is supported by grants PI18/00961 and PI21/00714 from 10.13039/501100004587Instituto de Salud Carlos III.

## Authors’ contributions

Study concept and design: DJP, NB, VM. Acquisition of data: TK, AF, PF, BM, EGG, FG, MAD, FAM, ADP, RDG, EC, PT, CA, VC, AUA, TM, SB, MB, NB, VM, AD. Analysis and interpretation of data: DJP, TK, PF, AD, FAM, NB, VM. Drafting of the manuscript: DJP, TK. Manuscript revision and input: all authors. Statistical analysis: DJP, TK, PF, AD. Obtained funding: DJP. Study supervision: DJP, VM.

## Data availability

The data that support the findings of this study are available from the corresponding author upon reasonable request.

## Conflicts of interest

DJP received lecture fees from ViiV Healthcare, Bayer Healthcare, BMS, Roche, Eisai, Falk Foundation, travel expenses from BMS and Bayer Healthcare; consulting fees for Mina Therapeutics, EISAI, Roche, DaVolterra, Mursla, LIfT Biosciences, Starpharma, Exact Sciences and Astra Zeneca; research funding (to institution) from MSD, GSK, and BMS. AF received lecture fees from Bayer HealthCare, Gilead, and MDS; Consulting fees from Bayer HealthCare, Roche, Guerbert, and Astra Zeneca. AD received educational support for congress attendance and consultancy fees from Roche. EG received lecture fees from Bayer HealthCare, Gilead, AbbVie, MSD, Eisai. JMM has received consulting honoraria and/or research grants from AbbVie, Angelini, Contrafect, Cubist, Genentech, Gilead Sciences, Jansen, Lysovant, Medtronic, MSD, Novartis, Pfizer, and ViiV Healthcare, outside the submitted work. NB received lecture fees from Abbvie and Gilead Sciences. BM received lecture fees from Eisai, MSD, Roche. Consultancy fees from Bayer-Shering Pharma, Eisai-Merck. All remaining authors have declared no conflicts of interest. The authors have no other relevant affiliations or financial involvement with any organisation or entity with a financial interest in or financial conflict with the subject matter or materials discussed in the manuscript apart from those disclosed. No writing assistance was utilised in the production of this manuscript.

Please refer to the accompanying ICMJE disclosure forms for further details.
