# Supplementary material for: Integrated phenotyping of the anti-cancer immune response in HIV-associated hepatocellular carcinoma
Source: JHEP Rep. 2023 Mar 22;5(7):100741. doi: 10.1016/j.jhepr.2023.100741 (PMC10238838; doi:10.1016/j.jhepr.2023.100741)
Supplement: Multimedia component 1 [file mmc1.pdf]

Integrated phenotyping of the anti-cancer immune response in HIV-associated hepatocellular carcinoma.

David J. Pinato Takahiro Kaneko, Antonio D'Alessio, Alejandro Forner, Petros Fessas, Beatriz Minguez, Edoardo G. Giannini, Federica Grillo, Alba Díaz, Francesco A. Mauri, Claudia A.M. Fulgenzi, Alessia Dalla Pria, Robert D. Goldin, Giulia Pieri, Pierluigi Toniutto, Claudio Avellini, Maria Corina Plaz Torres, Ayse U. Akarca, Teresa Marafioti, Sherrie Bhoori, Jose María Miró, Mark Bower, Norbert Bräu, Vincenzo Mazzaferro

Table of contents

FIGURES

Fig. S1. **Page 3.**

Kaplan-Meier curve illustrating the overall survival of patients with HCC stratified according to HIV status.

Fig. S2. **Page 4.**

The relationship between phenotypic characteristics of the intratumoral T-cell infiltrate and PD-L1 status in patients with HIV-associated HCC (n=63).

Fig. S3. **Page 5.**

Kaplan-Meier curve illustrating the overall survival of patients with HIV-associated HCC stratified according to PD-L1 TPS score (Positive if TPS$\geq$1; Negative if TPS<1).

Fig. S4. **Page 6.**

The relationship between phenotypic characteristics of the intratumoural T-cell infiltrate and PD-L1 status in patients with HIV-associated HCC (n=63).

Fig. S5. **Page 7.**

Heat map of the 770 transcripts analyzed with the Nanostring Pancancer Immune Panel in HIV-associated HCC compared with HIV-negative controls.

TABLES

Table S1. **Page 8.**
Patient disposition across participating institutions.

Table S2. **Page 9.**
Complete list of the targeted genes profiled using the NanoString PanCancer Immune panel.

Table S3. **Page 42.**
Functional categories and related numbers of genes profiled using the NanoString PanCancer Immune panel.

Table S4. **Page 43.**
Primary annotations and related number of genes profiled using the NanoString PanCancer Immune panel.

Table S5. **Page 46.**
Immune subtype and related number of genes profiled using the NanoString PanCancer Immune panel.

Table S6. **Page 47.**
Overall distribution of T-cell density in tumour and non-tumour background tissue across HIV+ (n=63) and HIV- patients (n=66).

Table S7. **Page 48.**
Overall distribution of T-cell density in tumour and non-tumour background tissue across Child-Turcotte-Pugh (CTP) A (n=79) and CTP B-C patients (n=48).

Table S8. **Page 49.**
Characteristics of patients who underwent transcriptomics and TCR sequencing analysis.

FIGURES

Fig. S1. Kaplan-Meier curve illustrating the overall survival of patients with HCC stratified according to HIV infection status.

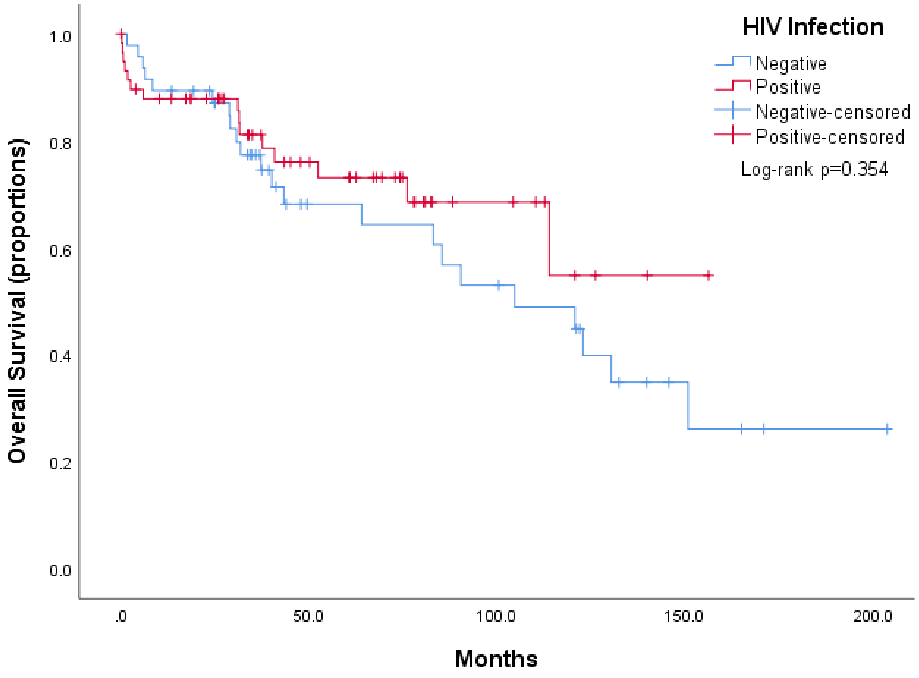

Fig. S2. The relationship between phenotypic characteristics of the intratumoral T-cell infiltrate and PD-L1 status in patients with HIV-associated HCC (n=63). TILs distribution was compared across PD-L1-positive and negative with Mann-Whitney U test. Statistical significance is reported as *. Abbreviations: ns, non significant; p, p value. P-values for associations: Panel A, p=0.399; Panel B, p=0.0493; Panel C, p=0.33; Panel D, p=0.26.

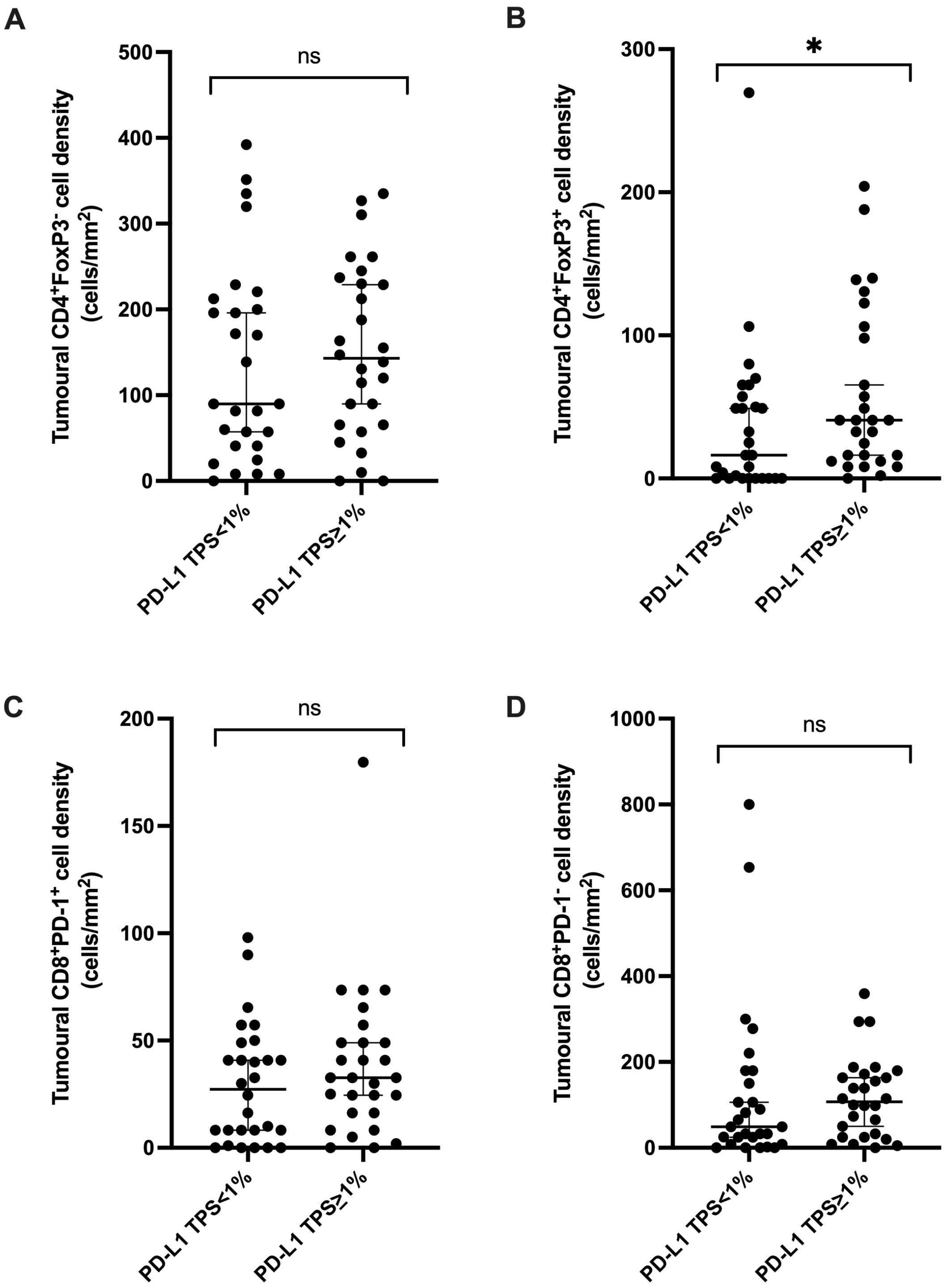

Fig. S3. Kaplan-Meier curve illustrating the overall survival of patients with HIV-associated HCC stratified according to PD-L1 TPS score (Positive if TPS$\geq$1; Negative if TPS<1).

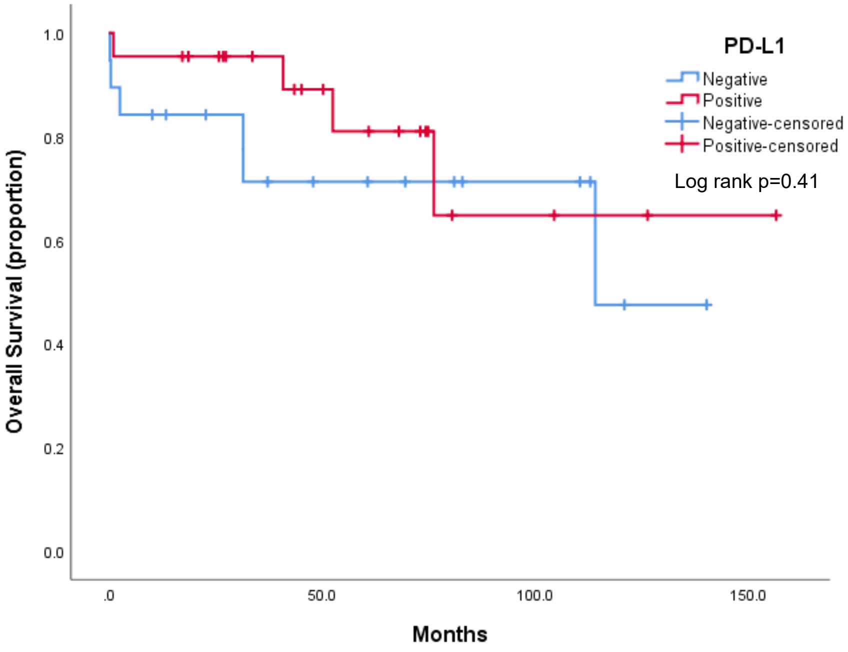

Fig. S4. The relationship between phenotypic characteristics of the intratumoural T-cell infiltrate and PD-L1 status in patients with HIV-associated HCC (n=63). For Panels A, F, G, and H, comparisons were made with Mann-Whitney U test. For Panels B, C, D, and E, correlation was assessed with Spearman's correlation coefficient test. Statistical significance is reported as *. Abbreviations: ns, non significant; p, p value.

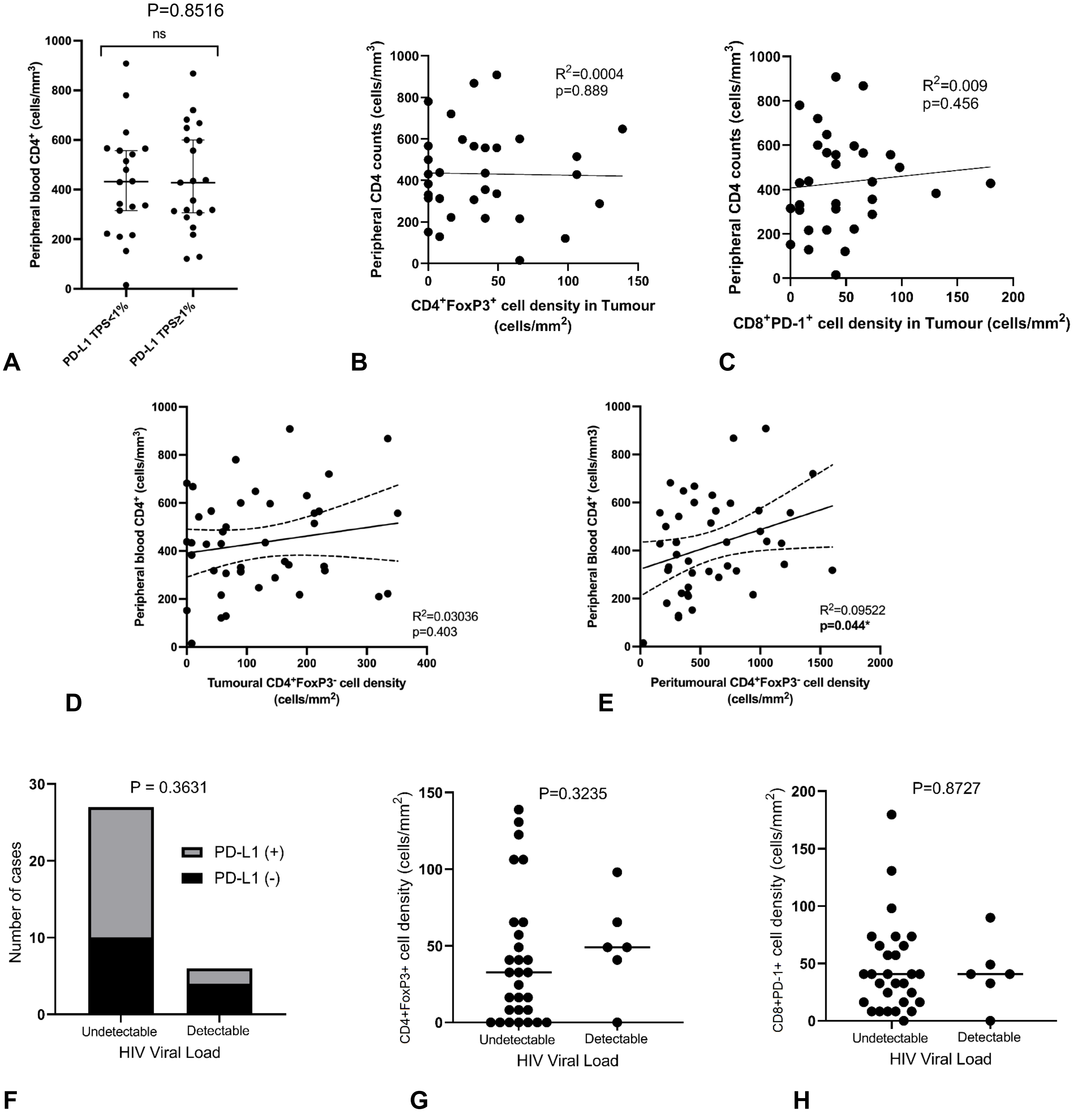

Fig. S5. Heat map of the 770 transcripts analyzed with the Nanostring Pancancer Immune Panel in HIV-associated HCC compared with HIV-negative controls.

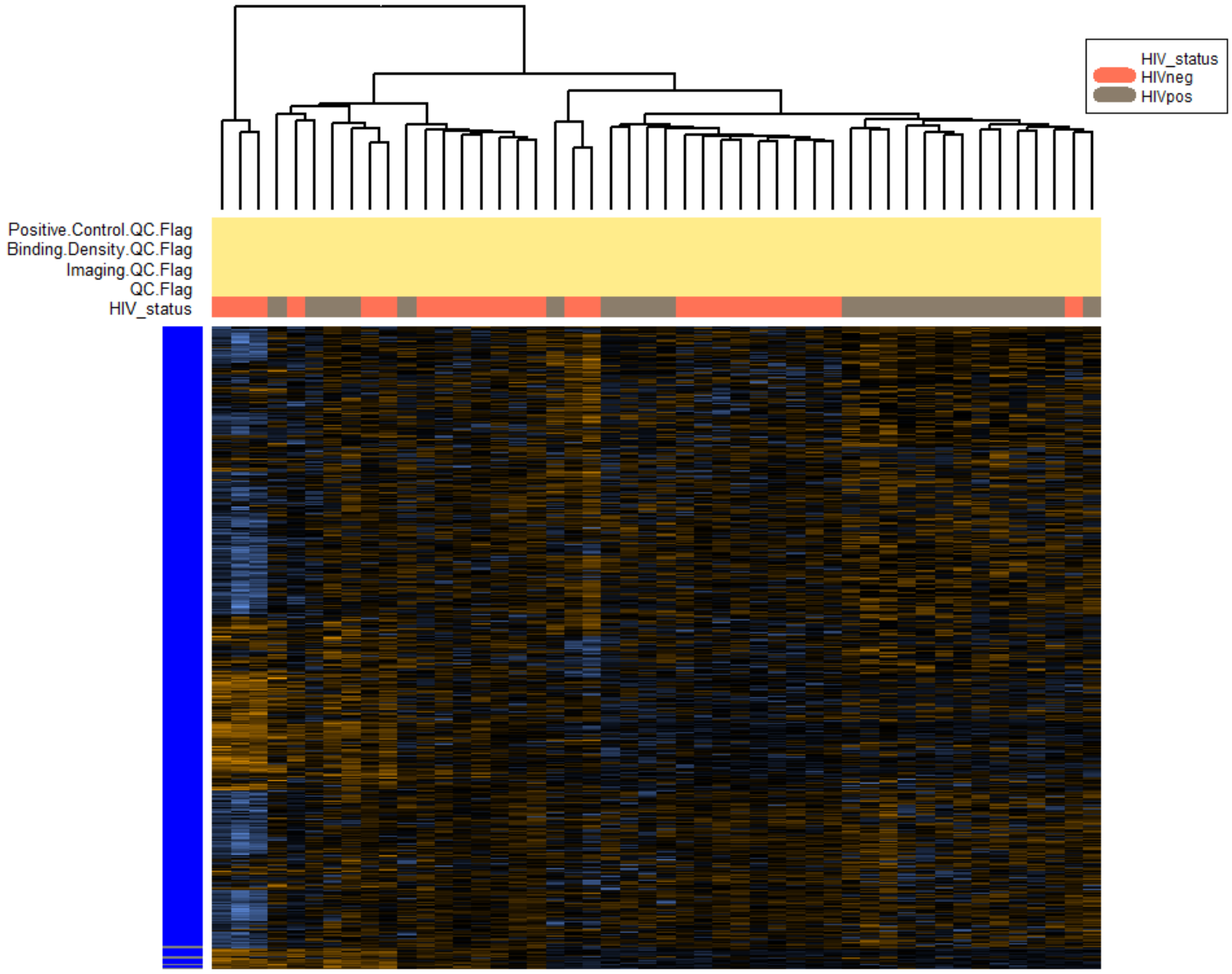

TABLES

Table S1. Patient disposition across participating institutions.

Participating Institution	Patient samples (n, %)
Imperial College London, London, United Kingdom	3 (2.3)
Chelsea and Westminster Hospital, London United Kingdom	2 (1.6)
Bronx VA Medical Centre, New York, United States of America	2 (1.6)
Istituto Nazionale Tumori, Milan, Italy	25 (19.4)
Hospital Clinic, Barcelona, Catalonia, Spain	18 (14.0)
Vall D'Hebron Hospital, Barcelona, Catalonia, Spain	6 (4.7)
San Martino University Hospital, Genova, Italy	10 (7.8)
University of Udine, Hepatology and Liver Transplantation Unit, Udine, Italy	63 (48.8)
**Total**	**129 (100)**

Table S2. Complete list of the targeted genes profiled using the NanoString PanCancer Immune panel, including the categories utilised for the Gene Set Analysis.

**Gene Name**	**Official Full Name**	**Immune Response Category for Gene Set Analysis**
A2M	alpha-2-macroglobulin	Chemokines
ABCB1	ATP-binding cassette, sub-family B (MDR/TAP), member 1	
ABL1	c-abl oncogene 1, non-receptor tyrosine kinase	Cell Cycle, Regulation, Senescence
ADA	adenosine deaminase	B-Cell Functions, T-Cell Functions
ADORA2A	adenosine A2a receptor	Cell Functions
AICDA	activation-induced cytidine deaminase	T-Cell Functions
AIRE	autoimmune regulator	
AKT3	v-akt murine thymoma viral oncogene homolog 3 (protein kinase B, gamma)	Cell Functions
ALCAM	activated leukocyte cell adhesion molecule	Adhesion
AMBP	alpha-1-microglobulin/bikunin precursor	Regulation
AMICA1	adhesion molecule, interacts with CXADR antigen 1	Regulation
ANP32B	acidic (leucine-rich) nuclear phosphoprotein 32 family, member B	Cell Functions
ANXA1	annexin A1	Transporter Functions
APOE	apolipoprotein E	Transporter Functions
APP	amyloid beta (A4) precursor protein	
ARG1	arginase, liver	
ARG2	arginase, type II	
ATF1	activating transcription factor 1	
ATF2	activating transcription factor 2	
ATG10	autophagy related 10	Transporter Functions
ATG12	autophagy related 12	

ATG16L1	autophagy related 16-like 1 (S. cerevisiae)	Transporter Functions
ATG5	autophagy related 5	
ATG7	autophagy related 7	Transporter Functions
ATM	ataxia telangiectasia mutated	Cell Cycle
AXL	AXL receptor tyrosine kinase	
BAGE	B melanoma antigen	
BATF	basic leucine zipper transcription factor, ATF-like	Cell Functions
BAX	BCL2-associated X protein	Cell Cycle, Regulation
BCL10	B-cell CLL/lymphoma 10	
BCL2	B-cell CLL/lymphoma 2	Cell Cycle
BCL2L1	BCL2-like 1	
BCL6	B-cell CLL/lymphoma 6	Regulation
BID	BH3 interacting domain death agonist	Cell Cycle, Regulation
BIRC5	baculoviral IAP repeat containing 5	Cell Cycle
BLK	B lymphoid tyrosine kinase	B-Cell Functions
BLNK	B-cell linker	
BMI1	BMI1 polycomb ring finger oncogene	
BST1	bone marrow stromal cell antigen 1	
BST2	bone marrow stromal cell antigen 2	
BTK	Bruton agammaglobulinemia tyrosine kinase	
BTLA	B and T lymphocyte associated	Cell Functions
C1QA	complement component 1, q subcomponent, A chain	Complement
C1QB	complement component 1, q subcomponent, B chain	Complement
C1QBP	complement component 1, q subcomponent binding protein	Chemokines
C1R	complement component 1, r subcomponent	Complement

C1S	complement component 1, s subcomponent	Complement
C2	complement component 2	Complement
C3	complement component 3	Regulation
C3AR1	complement component 3a receptor 1	Regulation
C4B	complement component 4B (Chido blood group)	Complement
C4BPA	complement component 4 binding protein, alpha	Complement
C5	complement component 5	Complement
C6	complement component 6	Complement
C7	complement component 7	Complement
C8A	complement component 8, alpha polypeptide	Complement
C8B	complement component 8, beta polypeptide	Complement
C8G	complement component 8, gamma polypeptide	Complement
C9	complement component 9	Complement
CAMP	cathelicidin antimicrobial peptide	
CARD11	caspase recruitment domain family, member 11	Regulation
CARD9	caspase recruitment domain family, member 9	
CASP1	caspase 1, apoptosis-related cysteine peptidase	
CASP10	caspase 10, apoptosis-related cysteine peptidase	
CASP3	caspase 3, apoptosis-related cysteine peptidase	Cell Cycle, Regulation
CASP8	caspase 8, apoptosis-related cysteine peptidase	
CCL1	chemokine (C-C motif) ligand 1	Chemokines
CCL11	chemokine (C-C motif) ligand 11	Chemokines
CCL13	chemokine (C-C motif) ligand 13	Chemokines
CCL14	chemokine (C-C motif) ligand 14	Chemokines
CCL15	chemokine (C-C motif) ligand 15	Chemokines

CCL16	chemokine (C-C motif) ligand 16	Chemokines, Regulation
CCL17	chemokine (C-C motif) ligand 17	Chemokines
CCL18	chemokine (C-C motif) ligand 18 (pulmonary and activation-regulated)	Chemokines
CCL19	chemokine (C-C motif) ligand 19	Chemokines, Regulation
CCL2	chemokine (C-C motif) ligand 2	Chemokines
CCL20	chemokine (C-C motif) ligand 20	Chemokines
CCL21	chemokine (C-C motif) ligand 21	Chemokines, Regulation
CCL22	chemokine (C-C motif) ligand 22	Chemokines, Pathogen Defense
CCL23	chemokine (C-C motif) ligand 23	Chemokines, Regulation
CCL24	chemokine (C-C motif) ligand 24	Chemokines, Regulation
CCL25	chemokine (C-C motif) ligand 25	Chemokines, Complement
CCL26	chemokine (C-C motif) ligand 26	Chemokines
CCL27	chemokine (C-C motif) ligand 27	Chemokines
CCL28	chemokine (C-C motif) ligand 28	Chemokines
CCL3	chemokine (C-C motif) ligand 3	Chemokines, Regulation
CCL3L1	chemokine (C-C motif) ligand 3-like 1	Cytokines
CCL4	chemokine (C-C motif) ligand 4	Chemokines, Regulation
CCL5	chemokine (C-C motif) ligand 5	Chemokines, Cytokines
CCL7	chemokine (C-C motif) ligand 7	Chemokines
CCL8	chemokine (C-C motif) ligand 8	Chemokines, Regulation
CCND3	cyclin D3	Cell Cycle
CCR1	chemokine (C-C motif) receptor 1	Chemokines, Cytokines, NK Cell Functions, Regulation, T-Cell Functions
CCR2	chemokine (C-C motif) receptor 2	Cytokines
CCR3	chemokine (C-C motif) receptor 3	Chemokines
CCR4	chemokine (C-C motif) receptor 4	Chemokines, Cytokines, Regulation, T-Cell Functions

CCR5	chemokine (C-C motif) receptor 5 (gene/pseudogene)	Cytokines, T-Cell Functions
CCR6	chemokine (C-C motif) receptor 6	
CCR7	chemokine (C-C motif) receptor 7	Chemokines, Regulation
CCR9	chemokine (C-C motif) receptor 9	
CCRL2	chemokine (C-C motif) receptor-like 2	Chemokines
CD14	CD14 molecule	
CD160	CD160 molecule	Regulation
CD163	CD163 molecule	Transporter Functions
CD164	CD164 molecule, sialomucin	
CD180	CD180 molecule	
CD19	CD19 molecule	B-Cell Functions, Regulation
CD1A	CD1a molecule	Cell Functions
CD1B	CD1b molecule	Cell Functions
CD1C	CD1c molecule	T-Cell Functions
CD1D	CD1d molecule	B-Cell Functions, Cell Functions, T-Cell Functions
CD1E	CD1e molecule	Antigen Processing
CD2	CD2 molecule	NK Cell Functions, T-Cell Functions
CD200	CD200 molecule	Regulation
CD207	CD207 molecule, langerin	
CD209	CD209 molecule	Cell Functions
CD22	CD22 molecule	
CD24	CD24 molecule	
CD244	CD244 molecule, natural killer cell receptor 2B4	
CD247	CD247 molecule	Regulation
CD27	CD27 molecule	B-Cell Functions, T-Cell Functions
CD274	CD274 molecule	B-Cell Functions, Cell Functions, T-Cell Functions

CD276	CD276 molecule	Regulation
CD28	CD28 molecule	
CD33	CD33 molecule	
CD34	CD34 molecule	Regulation
CD36	CD36 molecule (thrombospondin receptor)	Transporter Functions
CD37	CD37 molecule	
CD38	CD38 molecule	B-Cell Functions, Regulation, T-Cell Functions
CD3D	CD3d molecule, delta (CD3-TCR complex)	Regulation
CD3E	CD3e molecule, epsilon (CD3-TCR complex)	B-Cell Functions, Cell Functions, T-Cell Functions
CD3EAP	CD3e molecule, epsilon associated protein	Regulation
CD3G	CD3g molecule, gamma (CD3-TCR complex)	Regulation, T-Cell Functions
CD4	CD4 molecule	
CD40	CD40 molecule, TNF receptor superfamily member 5	Regulation
CD40LG	CD40 ligand	Regulation
CD44	CD44 molecule (Indian blood group)	Senescence, Transporter Functions
CD46	CD46 molecule, complement regulatory protein	
CD47	CD47 molecule	Macrophage Functions, Regulation, T-Cell Functions, Transporter Functions
CD48	CD48 molecule	
CD5	CD5 molecule	B-Cell Functions, Regulation, T-Cell Functions
CD53	CD53 molecule	
CD55	CD55 molecule, decay accelerating factor for complement (Cromer blood group)	
CD58	CD58 molecule	
CD59	CD59 molecule, complement regulatory protein	
CD6	CD6 molecule	Cell Functions
CD63	CD63 molecule	

CD68	CD68 molecule	Cell Functions
CD7	CD7 molecule	NK Cell Functions, Regulation, T-Cell Functions
CD70	CD70 molecule	B-Cell Functions, Cell Functions, Cytokines, T-Cell Functions, TNF Superfamily
CD74	CD74 molecule, major histocompatibility complex, class II invariant chain	
CD79A	CD79a molecule, immunoglobulin-associated alpha	
CD79B	CD79b molecule, immunoglobulin-associated beta	B-Cell Functions
CD80	CD80 molecule	B-Cell Functions, Macrophage Functions, Regulation, T-Cell Functions
CD81	CD81 molecule	Regulation
CD83	CD83 molecule	
CD84	CD84 molecule	
CD86	CD86 molecule	B-Cell Functions, Macrophage Functions, Regulation, T-Cell Functions
CD8A	CD8a molecule	Antigen Processing, Pathogen Defense, T-Cell Functions
CD8B	CD8b molecule	Regulation, T-Cell Functions
CD9	CD9 molecule	
CD96	CD96 molecule	Regulation
CD97	CD97 molecule	
CD99	CD99 molecule	
CDH1	cadherin 1, type 1, E-cadherin (epithelial)	Regulation
CDH5	cadherin 5, type 2 (vascular endothelium)	
CDK1	cyclin-dependent kinase 1	
CDKN1A	cyclin-dependent kinase inhibitor 1A (p21, Cip1)	Cell Cycle, Regulation, Senescence
CEACAM1	carcinoembryonic antigen-related cell adhesion molecule 1 (biliary glycoprotein)	Adhesion

CEACAM6	carcinoembryonic antigen-related cell adhesion molecule 6 (non-specific cross reacting antigen)	Adhesion
CEACAM8	carcinoembryonic antigen-related cell adhesion molecule 8	Adhesion, Chemokines
CEBPB	CCAAT/enhancer binding protein (C/EBP), beta	
CFB	complement factor B	
CFD	complement factor D (adipsin)	
CFI	complement factor I	
CFP	complement factor properdin	
CHIT1	chitinase 1 (chitotriosidase)	Cell Functions
CHUK	conserved helix-loop-helix ubiquitous kinase	
CKLF	chemokine-like factor	Chemokines
CLEC4A	C-type lectin domain family 4, member A	
CLEC4C	C-type lectin domain family 4, member C	
CLEC5A	C-type lectin domain family 5, member A	
CLEC6A	C-type lectin domain family 6, member A	
CLEC7A	C-type lectin domain family 7, member A	
CLU	clusterin	
CMA1	chymase 1, mast cell	Regulation
CMKLR1	chemokine-like receptor 1	Chemokines
COL3A1	collagen, type III, alpha 1	Regulation
COLEC12	collectin sub-family member 12	
CR1	complement component (3b/4b) receptor 1 (Knops blood group)	
CR2	complement component (3d/Epstein Barr virus) receptor 2	B-Cell Functions
CREB1	cAMP responsive element binding protein 1	
CREB5	cAMP responsive element binding protein 5	

CREBBP	CREB binding protein	
CRP	C-reactive protein, pentraxin-related	Transporter Functions
CSF1	colony stimulating factor 1 (macrophage)	
CSF1R	colony stimulating factor 1 receptor	
CSF2	colony stimulating factor 2 (granulocyte-macrophage)	Cytokines, Macrophage Functions, Regulation
CSF2RB	colony stimulating factor 2 receptor, beta, low-affinity (granulocyte-macrophage)	Chemokines
CSF3	colony stimulating factor 3 (granulocyte)	Cell Functions
CSF3R	colony stimulating factor 3 receptor (granulocyte)	Cytokines
CT45A1	cancer/testis antigen family 45, member A1	
CTAG1B	cancer/testis antigen 1B	
CTAGE1	cutaneous T-cell lymphoma-associated antigen 1	
CTCFL	CCCTC-binding factor (zinc finger protein)-like	
CTLA4	cytotoxic T-lymphocyte-associated protein 4	B-Cell Functions, T-Cell Functions
CTSG	cathepsin G	Pathogen Defense, Regulation
CTSH	cathepsin H	
CTSL	cathepsin L	
CTSS	cathepsin S	
CTSW	cathepsin W	Transporter Functions
CX3CL1	chemokine (C-X3-C motif) ligand 1	Chemokines, Leukocyte Functions
CX3CR1	chemokine (C-X3-C motif) receptor 1	Chemokines, Microglial Functions
CXCL1	chemokine (C-X-C motif) ligand 1 (melanoma growth stimulating activity, alpha)	Chemokines, Regulation
CXCL10	chemokine (C-X-C motif) ligand 10	Chemokines, Cytokines, Pathogen Defense, Regulation, T-Cell Functions
CXCL11	chemokine (C-X-C motif) ligand 11	Chemokines, NK Cell Functions, T-Cell Functions

CXCL12	chemokine (C-X-C motif) ligand 12	Chemokines
CXCL13	chemokine (C-X-C motif) ligand 13	Chemokines
CXCL14	chemokine (C-X-C motif) ligand 14	Chemokines
CXCL16	chemokine (C-X-C motif) ligand 16	Chemokines
CXCL2	chemokine (C-X-C motif) ligand 2	Chemokines, Regulation
CXCL3	chemokine (C-X-C motif) ligand 3	Chemokines, Regulation
CXCL5	chemokine (C-X-C motif) ligand 5	Chemokines
CXCL6	chemokine (C-X-C motif) ligand 6 (granulocyte chemotactic protein 2)	Chemokines, Regulation
CXCL9	chemokine (C-X-C motif) ligand 9	Chemokines, Regulation, T-Cell Functions
CXCR1	chemokine (C-X-C motif) receptor 1	Chemokines, Regulation
CXCR2	chemokine (C-X-C motif) receptor 2	Chemokines, Regulation
CXCR3	chemokine (C-X-C motif) receptor 3	Chemokines, NK Cell Functions, T-Cell Functions
CXCR4	chemokine (C-X-C motif) receptor 4	Cell Cycle, Cell Functions, Chemokines, Regulation
CXCR5	chemokine (C-X-C motif) receptor 5	B-Cell Functions, Chemokines, T-Cell Functions
CXCR6	chemokine (C-X-C motif) receptor 6	Chemokines
CYBB	cytochrome b-245, beta polypeptide	
CYFIP2	cytoplasmic FMR1 interacting protein 2	
CYLD	cylindromatosis (turban tumor syndrome)	
DDX43	DEAD (Asp-Glu-Ala-Asp) box polypeptide 43	
DDX58	DEAD (Asp-Glu-Ala-Asp) box polypeptide 58	
DEFB1	defensin, beta 1	
DMBT1	deleted in malignant brain tumors 1	
DOCK9	dedicator of cytokinesis 9	Cell Functions
DPP4	dipeptidyl-peptidase 4	Macrophage Functions, Regulation, T-Cell Functions
DUSP4	dual specificity phosphatase 4	

DUSP6	dual specificity phosphatase 6	
EBI3	Epstein-Barr virus induced 3	Cytokines
ECSIT	ECSIT homolog (Drosophila)	
EGR1	early growth response 1	Senescence, T-Cell Functions
EGR2	early growth response 2	Regulation
ELANE	elastase, neutrophil expressed	Regulation
ELK1	ELK1, member of ETS oncogene family	
ENG	endoglin	
ENTPD1	ectonucleoside triphosphate diphosphohydrolase 1	
EOMES	eomesodermin	T-Cell Functions
EP300	E1A binding protein p300	
EPCAM	epithelial cell adhesion molecule	Adhesion, Cell Functions
ETS1	v-ets erythroblastosis virus E26 oncogene homolog 1 (avian)	Senescence
EWSR1	Ewing sarcoma breakpoint region 1	Cell Functions
F12	coagulation factor XII (Hageman factor)	
F13A1	coagulation factor XIII, A1 polypeptide	Cell Functions
F2RL1	coagulation factor II (thrombin) receptor-like 1	Macrophage Functions, T-Cell Functions
FADD	Fas (TNFRSF6)-associated via death domain	
FAS	Fas (TNF receptor superfamily, member 6)	B-Cell Functions, Regulation, T-Cell Functions, TNF Superfamily, Transporter Functions
FCER1A	Fc fragment of IgE, high affinity I, receptor for; alpha polypeptide	
FCER1G	Fc fragment of IgE, high affinity I, receptor for; gamma polypeptide	Regulation
FCER2	Fc fragment of IgE, low affinity II, receptor for (CD23)	
FCGR1A	Fc fragment of IgG, high affinity Ia, receptor (CD64)	
FCGR2A	Fc fragment of IgG, low affinity IIa, receptor (CD32)	Transporter Functions

FCGR2B	Fc fragment of IgG, low affinity IIb, receptor (CD32)	Regulation
FCGR3A	Fc fragment of IgG, low affinity IIIa, receptor (CD16a)	Regulation
FEZ1	fasciculation and elongation protein zeta 1 (zygin I)	Cell Functions
FLT3	fms-related tyrosine kinase 3	
FLT3LG	fms-related tyrosine kinase 3 ligand	Cytokines
FN1	fibronectin 1	Senescence
FOS	FBJ murine osteosarcoma viral oncogene homolog	
FOXJ1	forkhead box J1	
FOXP3	forkhead box P3	Cytokines, T-Cell Functions
FPR2	formyl peptide receptor 2	
FUT5	fucosyltransferase 5 (alpha (1,3) fucosyltransferase)	Cell Functions
FUT7	fucosyltransferase 7 (alpha (1,3) fucosyltransferase)	Leukocyte Functions
FYN	FYN oncogene related to SRC, FGR, YES	Transporter Functions
GAGE1	G antigen 1	
GATA3	GATA binding protein 3	Cell Functions
GNLY	granulysin	Cytotoxicity, Pathogen Defense
GPI	glucose-6-phosphate isomerase	
GTF3C1	general transcription factor IIIC, polypeptide 1, alpha 220kDa	Cell Functions
GZMA	granzyme A (granzyme 1, cytotoxic T-lymphocyte-associated serine esterase 3)	Cell Functions, Cytotoxicity
GZMB	granzyme B (granzyme 2, cytotoxic T-lymphocyte-associated serine esterase 1)	Cell Functions, Cytotoxicity
GZMH	granzyme H (cathepsin G-like 2, protein h-CCPX)	Cell Functions, Cytotoxicity
GZMK	granzyme K (granzyme 3; tryptase II)	Cell Functions, Cytotoxicity
GZMM	granzyme M (lymphocyte met-ase 1)	Cell Functions, Cytotoxicity
HAMP	hepcidin antimicrobial peptide	

HAVCR2	hepatitis A virus cellular receptor 2	Cell Functions
HCK	hemopoietic cell kinase	Leukocyte Functions
HLA-A	major histocompatibility complex, class I, A	Antigen Processing, Cytotoxicity, Regulation
HLA-B	major histocompatibility complex, class I, B	Antigen Processing, Cytotoxicity, Regulation
HLA-C	major histocompatibility complex, class I, C	Antigen Processing, Cytotoxicity, Regulation
HLA-DMA	major histocompatibility complex, class II, DM alpha	Antigen Processing, Regulation
HLA-DMB	major histocompatibility complex, class II, DM beta	Antigen Processing
HLA-DOB	major histocompatibility complex, class II, DO beta	Antigen Processing, Cytokines
HLA-DPA1	major histocompatibility complex, class II, DP alpha 1	Antigen Processing
HLA-DPB1	major histocompatibility complex, class II, DP beta 1	Antigen Processing
HLA-DQA1	major histocompatibility complex, class II, DQ alpha 1	Antigen Processing
HLA-DQB1	major histocompatibility complex, class II, DQ beta 1	Antigen Processing
HLA-DRA	major histocompatibility complex, class II, DR alpha	Antigen Processing
HLA-DRB3	major histocompatibility complex, class II, DR beta 3	Antigen Processing
HLA-DRB4	major histocompatibility complex, class II, DR beta 4	Antigen Processing
HLA-E	major histocompatibility complex, class I, E	Regulation
HLA-G	major histocompatibility complex, class I, G	Regulation
HMGB1	high mobility group box 1	Regulation
HRAS	v-Ha-ras Harvey rat sarcoma viral oncogene homolog	Senescence
HSD11B1	hydroxysteroid (11-beta) dehydrogenase 1	Cell Functions
ICAM1	intercellular adhesion molecule 1	Adhesion, Regulation
ICAM2	intercellular adhesion molecule 2	Adhesion, Regulation
ICAM3	intercellular adhesion molecule 3	Adhesion, Regulation
ICAM4	intercellular adhesion molecule 4 (Landsteiner-Wiener blood group)	Adhesion, Regulation

ICOS	inducible T-cell co-stimulator	Cell Functions
ICOSLG	inducible T-cell co-stimulator ligand	Cell Functions, Regulation
IDO1	indoleamine 2,3-dioxygenase 1	Cytokines, T-Cell Functions
IFI16	interferon, gamma-inducible protein 16	Chemokines
IFI27	interferon, alpha-inducible protein 27	Chemokines
IFI35	interferon-induced protein 35	Chemokines
IFIH1	interferon induced with helicase C domain 1	
IFIT1	interferon-induced protein with tetratricopeptide repeats 1	Chemokines
IFIT2	interferon-induced protein with tetratricopeptide repeats 2	Chemokines
IFITM1	interferon induced transmembrane protein 1	Regulation
IFITM2	interferon induced transmembrane protein 2	
IFNA1	interferon, alpha 1	Interleukins
IFNA17	interferon, alpha 17	Interleukins
IFNA2	interferon, alpha 2	Interleukins
IFNA7	interferon, alpha 7	Interleukins
IFNA8	interferon, alpha 8	Interleukins
IFNAR1	interferon (alpha, beta and omega) receptor 1	Pathogen Defense
IFNAR2	interferon (alpha, beta and omega) receptor 2	Chemokines
IFNB1	interferon, beta 1, fibroblast	
IFNG	interferon, gamma	Cytokines, Interleukins, Leukocyte Functions, Macrophage Functions, NK Cell Functions, T-Cell Functions
IFNGR1	interferon gamma receptor 1	Cell Functions
IFNL1	interferon lambda 1	Cytokines, Interleukins
IFNL2	interferon lambda 2	Chemokines
IGF1R	insulin-like growth factor 1 receptor	Senescence
IGF2R	insulin-like growth factor 2 receptor	

IGLL1	immunoglobulin lambda-like polypeptide 1	
IKBKB	inhibitor of kappa light polypeptide gene enhancer in B-cells, kinase beta	
IKBKE	inhibitor of kappa light polypeptide gene enhancer in B-cells, kinase epsilon	
IKBKG	inhibitor of kappa light polypeptide gene enhancer in B-cells, kinase gamma	
IL10	interleukin 10	Interleukins
IL10RA	interleukin 10 receptor, alpha	Cytokines
IL11	interleukin 11	B-Cell Functions, Cytokines, Interleukins, T-Cell Functions
IL11RA	interleukin 11 receptor, alpha	Chemokines
IL12A	interleukin 12A (natural killer cell stimulatory factor 1, cytotoxic lymphocyte maturation factor 1, p35)	Cytokines, Interleukins, NK Cell Functions, Regulation, T-Cell Functions
IL12B	interleukin 12B (natural killer cell stimulatory factor 2, cytotoxic lymphocyte maturation factor 2, p40)	Cytokines, Interleukins, NK Cell Functions, T-Cell Functions
IL12RB1	interleukin 12 receptor, beta 1	NK Cell Functions, T-Cell Functions
IL12RB2	interleukin 12 receptor, beta 2	Cytokines, NK Cell Functions, T-Cell Functions
IL13	interleukin 13	Cytokines, Interleukins, T-Cell Functions
IL13RA1	interleukin 13 receptor, alpha 1	Cytokines, T-Cell Functions
IL13RA2	interleukin 13 receptor, alpha 2	Chemokines, T-Cell Functions
IL15	interleukin 15	Interleukins, Regulation
IL15RA	interleukin 15 receptor, alpha	Chemokines
IL16	interleukin 16	Interleukins
IL17A	interleukin 17A	Cytokines, Interleukins
IL17B	interleukin 17B	Chemokines, Interleukins
IL17F	interleukin 17F	Interleukins
IL17RA	interleukin 17 receptor A	Cell Functions
IL17RB	interleukin 17 receptor B	Chemokines

IL18	interleukin 18 (interferon-gamma-inducing factor)	Interleukins, NK Cell Functions, T-Cell Functions
IL18R1	interleukin 18 receptor 1	NK Cell Functions, T-Cell Functions
IL18RAP	interleukin 18 receptor accessory protein	NK Cell Functions, T-Cell Functions
IL19	interleukin 19	Chemokines, Interleukins
IL1A	interleukin 1, alpha	Cytokines, Interleukins
IL1B	interleukin 1, beta	Chemokines, Cytokines, Interleukins, Pathogen Defense, Regulation
IL1R1	interleukin 1 receptor, type I	
IL1R2	interleukin 1 receptor, type II	Cytokines
IL1RAP	interleukin 1 receptor accessory protein	
IL1RAPL2	interleukin 1 receptor accessory protein-like 2	
IL1RL1	interleukin 1 receptor-like 1	
IL1RL2	interleukin 1 receptor-like 2	
IL1RN	interleukin 1 receptor antagonist	Cytokines, Interleukins
IL2	interleukin 2	Cytokines, Regulation, T-Cell Functions
IL21	interleukin 21	Cytokines, Interleukins
IL21R	interleukin 21 receptor	Cell Functions
IL22	interleukin 22	Cytokines
IL22RA1	interleukin 22 receptor, alpha 1	Chemokines
IL22RA2	interleukin 22 receptor, alpha 2	Chemokines
IL23A	interleukin 23, alpha subunit p19	Interleukins
IL23R	interleukin 23 receptor	Cytokines
IL24	interleukin 24	Cytokines, Interleukins
IL25	interleukin 25	Interleukins
IL26	interleukin 26	Cytokines, Interleukins
IL27	interleukin 27	Interleukins
IL2RA	interleukin 2 receptor, alpha	Regulation

IL2RB	interleukin 2 receptor, beta	Cytokines
IL2RG	interleukin 2 receptor, gamma	Chemokines
IL3	interleukin 3 (colony-stimulating factor, multiple)	Regulation, T-Cell Functions
IL32	interleukin 32	Chemokines, Interleukins
IL34	interleukin 34	Interleukins
IL3RA	interleukin 3 receptor, alpha (low affinity)	Cell Functions
IL4	interleukin 4	Interleukins, Regulation, T-Cell Functions
IL4R	interleukin 4 receptor	Cytokines, T-Cell Functions
IL5	interleukin 5 (colony-stimulating factor, eosinophil)	Cytokines, Interleukins, Regulation, T-Cell Functions
IL5RA	interleukin 5 receptor, alpha	Cytokines
IL6	interleukin 6 (interferon, beta 2)	Interleukins
IL6R	interleukin 6 receptor	Cytokines
IL6ST	interleukin 6 signal transducer (gp130, oncostatin M receptor)	Chemokines
IL7	interleukin 7	Interleukins
IL7R	interleukin 7 receptor	Cytokines
IL8	C-X-C motif chemokine ligand 8	Chemokines, Cytokines, Interleukins, Pathogen Defense, Regulation
IL9	interleukin 9	Cytokines
ILF3	interleukin enhancer binding factor 3, 90kDa	Chemokines
INPP5D	inositol polyphosphate-5-phosphatase, 145kDa	Regulation
IRAK1	interleukin-1 receptor-associated kinase 1	
IRAK2	interleukin-1 receptor-associated kinase 2	
IRAK4	interleukin-1 receptor-associated kinase 4	
IRF1	interferon regulatory factor 1	Chemokines, NK Cell Functions, Regulation, T-Cell Functions
IRF2	interferon regulatory factor 2	Chemokines, Regulation
IRF3	interferon regulatory factor 3	

IRF4	interferon regulatory factor 4	B-Cell Functions, Regulation, T-Cell Functions
IRF5	interferon regulatory factor 5	Senescence
IRF7	interferon regulatory factor 7	
IRF8	interferon regulatory factor 8	Chemokines, Regulation
IRGM	immunity-related GTPase family, M	
ISG15	ISG15 ubiquitin-like modifier	
ISG20	interferon stimulated exonuclease gene 20kDa	
ITCH	itchy E3 ubiquitin protein ligase	
ITGA1	integrin, alpha 1	Adhesion, NK Cell Functions, T-Cell Functions
ITGA2	integrin, alpha 2 (CD49B, alpha 2 subunit of VLA-2 receptor)	Adhesion
ITGA2B	integrin, alpha 2b (platelet glycoprotein IIb of IIb/IIIa complex, antigen CD41)	Adhesion
ITGA4	integrin, alpha 4 (antigen CD49D, alpha 4 subunit of VLA-4 receptor)	Adhesion, Regulation
ITGA5	integrin, alpha 5 (fibronectin receptor, alpha polypeptide)	Adhesion
ITGA6	integrin, alpha 6	Adhesion
ITGAE	integrin, alpha E (antigen CD103, human mucosal lymphocyte antigen 1; alpha polypeptide)	Adhesion
ITGAL	integrin, alpha L (antigen CD11A (p180), lymphocyte function-associated antigen 1; alpha polypeptide)	Adhesion, Regulation
ITGAM	integrin, alpha M (complement component 3 receptor 3 subunit)	Adhesion, Transporter Functions
ITGAX	integrin, alpha X (complement component 3 receptor 4 subunit)	Adhesion
ITGB1	integrin, beta 1 (fibronectin receptor, beta polypeptide, antigen CD29 includes MDF2, MSK12)	Adhesion, Regulation
ITGB2	integrin, beta 2 (complement component 3 receptor 3 and 4 subunit)	Adhesion, Regulation
ITGB3	integrin, beta 3 (platelet glycoprotein IIIa, antigen CD61)	Adhesion

ITGB4	integrin, beta 4	Adhesion
ITK	IL2-inducible T-cell kinase	
JAK1	Janus kinase 1	Cytokines, Regulation
JAK2	Janus kinase 2	Cytokines, Regulation
JAK3	Janus kinase 3	Cytokines, Regulation
JAM3	junctional adhesion molecule 3	
KIR_Activating_Subgroup_1	killer cell immunoglobulin-like receptor, three domains, short cytoplasmic tail, 1	NK Cell Functions, Regulation
KIR_Activating_Subgroup_2	killer cell immunoglobulin-like receptor, two domains, short cytoplasmic tail, 1	NK Cell Functions, Regulation
KIR_Inhibiting_Subgroup_1	killer cell immunoglobulin-like receptor, two domains, long cytoplasmic tail, 1	NK Cell Functions, Regulation
KIR_Inhibiting_Subgroup_2	killer cell immunoglobulin-like receptor, two domains, long cytoplasmic tail, 3	NK Cell Functions, Regulation
KIR3DL1	killer cell immunoglobulin-like receptor, three domains, long cytoplasmic tail, 1	NK Cell Functions, Regulation
KIR3DL2	killer cell immunoglobulin-like receptor, three domains, long cytoplasmic tail, 2	NK Cell Functions, Regulation
KIR3DL3	killer cell immunoglobulin-like receptor, three domains, long cytoplasmic tail, 3	NK Cell Functions, Regulation
KIT	v-kit Hardy-Zuckerman 4 feline sarcoma viral oncogene homolog	Cell Functions
KLRB1	killer cell lectin-like receptor subfamily B, member 1	Chemokines, NK Cell Functions
KLRC1	killer cell lectin-like receptor subfamily C, member 1	NK Cell Functions, Regulation
KLRC2	killer cell lectin-like receptor subfamily C, member 2	NK Cell Functions
KLRD1	killer cell lectin-like receptor subfamily D, member 1	NK Cell Functions, Regulation
KLRF1	killer cell lectin-like receptor subfamily F, member 1	Cell Functions, NK Cell Functions
KLRG1	killer cell lectin-like receptor subfamily G, member 1	NK Cell Functions, Regulation

KLRK1	killer cell lectin-like receptor subfamily K, member 1	NK Cell Functions, Regulation
LAG3	lymphocyte-activation gene 3	Regulation, T-Cell Functions
LAIR2	leukocyte-associated immunoglobulin-like receptor 2	Cell Functions
LAMP1	lysosomal-associated membrane protein 1	Transporter Functions
LAMP2	lysosomal-associated membrane protein 2	
LAMP3	lysosomal-associated membrane protein 3	Cell Functions
LBP	lipopolysaccharide binding protein	Macrophage Functions
LCK	lymphocyte-specific protein tyrosine kinase	Regulation, T-Cell Functions
LCN2	lipocalin 2	
LCP1	lymphocyte cytosolic protein 1 (L-plastin)	Leukocyte Functions, Macrophage Functions, T-Cell Functions
LGALS3	lectin, galactoside-binding, soluble, 3	
LIF	leukemia inhibitory factor	Cell Functions
LILRA1	leukocyte immunoglobulin-like receptor, subfamily A (with TM domain), member 1	Regulation
LILRA4	leukocyte immunoglobulin-like receptor, subfamily A (with TM domain), member 4	
LILRA5	leukocyte immunoglobulin-like receptor, subfamily A (with TM domain), member 5	
LILRB1	leukocyte immunoglobulin-like receptor, subfamily B (with TM and ITIM domains), member 1	NK Cell Functions, Regulation, T-Cell Functions
LILRB2	leukocyte immunoglobulin-like receptor, subfamily B (with TM and ITIM domains), member 2	Regulation
LILRB3	leukocyte immunoglobulin-like receptor, subfamily B (with TM and ITIM domains), member 3	Regulation
LRP1	low density lipoprotein receptor-related protein 1	
LRRN3	leucine rich repeat neuronal 3	
LTA	lymphotoxin alpha (TNF superfamily, member 1)	Chemokines
LTB	lymphotoxin beta (TNF superfamily, member 3)	Cytokines, TNF Superfamily

LTBR	lymphotoxin beta receptor (TNFR superfamily, member 3)	Chemokines
LTF	lactotransferrin	
LTK	leukocyte receptor tyrosine kinase	Cell Functions
LY86	lymphocyte antigen 86	
LY9	lymphocyte antigen 9	
LY96	lymphocyte antigen 96	
LYN	v-yes-1 Yamaguchi sarcoma viral related oncogene homolog	Regulation
MAF	v-maf musculoaponeurotic fibrosarcoma oncogene homolog (avian)	Cell Functions, T-Cell Functions
MAGEA1	melanoma antigen family A, 1 (directs expression of antigen MZ2-E)	
MAGEA12	melanoma antigen family A, 12	
MAGEA3	melanoma antigen family A, 3	
MAGEA4	melanoma antigen family A, 4	
MAGEB2	melanoma antigen family B, 2	
MAGEC1	melanoma antigen family C, 1	
MAGEC2	melanoma antigen family C, 2	
MAP2K1	mitogen-activated protein kinase kinase 1	
MAP2K2	mitogen-activated protein kinase kinase 2	
MAP2K4	mitogen-activated protein kinase kinase 4	
MAP3K1	mitogen-activated protein kinase kinase kinase 1, E3 ubiquitin protein ligase	
MAP3K5	mitogen-activated protein kinase kinase kinase 5	
MAP3K7	mitogen-activated protein kinase kinase kinase 7	
MAP4K2	mitogen-activated protein kinase kinase kinase kinase 2	
MAPK1	mitogen-activated protein kinase 1	
MAPK11	mitogen-activated protein kinase 11	

MAPK14	mitogen-activated protein kinase 14	
MAPK3	mitogen-activated protein kinase 3	
MAPK8	mitogen-activated protein kinase 8	
MAPKAPK2	mitogen-activated protein kinase-activated protein kinase 2	
MARCO	macrophage receptor with collagenous structure	
MASP1	mannan-binding lectin serine peptidase 1 (C4/C2 activating component of Ra-reactive factor)	
MASP2	mannan-binding lectin serine peptidase 2	
MAVS	mitochondrial antiviral signaling protein	
MBL2	mannose-binding lectin (protein C) 2, soluble	
MCAM	melanoma cell adhesion molecule	Adhesion
MEF2C	myocyte enhancer factor 2C	
MEFV	Mediterranean fever	
MERTK	c-mer proto-oncogene tyrosine kinase	Transporter Functions
MFGE8	milk fat globule-EGF factor 8 protein	Transporter Functions
MICA	MHC class I polypeptide-related sequence A	Regulation
MICB	MHC class I polypeptide-related sequence B	Regulation
MIF	macrophage migration inhibitory factor (glycosylation-inhibiting factor)	
MME	membrane metallo-endopeptidase	Cell Functions
MNX1	motor neuron and pancreas homeobox 1	
MPPED1	metallophosphoesterase domain containing 1	Cell Functions
MR1	major histocompatibility complex, class I-related	Antigen Processing
MRC1	mannose receptor, C type 1	
MS4A1	membrane-spanning 4-domains, subfamily A, member 1	B-Cell Functions
MS4A2	membrane-spanning 4-domains, subfamily A, member 2	Chemokines

MSR1	macrophage scavenger receptor 1	Cell Functions
MST1R	macrophage stimulating 1 receptor (c-met-related tyrosine kinase)	
MUC1	mucin 1, cell surface associated	
MX1	myxovirus (influenza virus) resistance 1, interferon-inducible protein p78 (mouse)	
MYD88	myeloid differentiation primary response gene (88)	TLR
NCAM1	neural cell adhesion molecule 1	
NCF4	neutrophil cytosolic factor 4, 40kDa	
NCR1	natural cytotoxicity triggering receptor 1	Cell Functions, NK Cell Functions
NEFL	neurofilament, light polypeptide	Cell Functions
NFATC1	nuclear factor of activated T-cells, cytoplasmic, calcineurin-dependent 1	Regulation
NFATC2	nuclear factor of activated T-cells, cytoplasmic, calcineurin-dependent 2	Regulation
NFATC3	nuclear factor of activated T-cells, cytoplasmic, calcineurin-dependent 3	Regulation
NFATC4	nuclear factor of activated T-cells, cytoplasmic, calcineurin-dependent 4	
NFKB1	nuclear factor of kappa light polypeptide gene enhancer in B-cells 1	
NFKB2	nuclear factor of kappa light polypeptide gene enhancer in B-cells 2 (p49/p100)	
NFKBIA	nuclear factor of kappa light polypeptide gene enhancer in B-cells inhibitor, alpha	
NLRC5	NLR family, CARD domain containing 5	
NLRP3	NLR family, pyrin domain containing 3	
NOD1	nucleotide-binding oligomerization domain containing 1	
NOD2	nucleotide-binding oligomerization domain containing 2	Cytokines
NOS2A	nitric oxide synthase 2	
NOTCH1	notch 1	Regulation
NRP1	neuropilin 1	Cell Functions

NT5E	5'-nucleotidase, ecto (CD73)	Transporter Functions
NUP107	nucleoporin 107kDa	Cell Cycle
OAS3	2'-5'-oligoadenylate synthetase 3, 100kDa	Cytokines, Pathogen Defense
OSM	oncostatin M	Cell Functions
PASD1	PAS domain containing 1	
PAX5	paired box 5	
PBK	PDZ binding kinase	
PDCD1	programmed cell death 1	Regulation
PDCD1LG2	programmed cell death 1 ligand 2	
PDGFC	platelet derived growth factor C	Cell Functions
PDGFRB	platelet-derived growth factor receptor, beta polypeptide	
PECAM1	platelet/endothelial cell adhesion molecule 1	Transporter Functions
PIK3CD	phosphatidylinositol-4,5-bisphosphate 3-kinase, catalytic subunit delta	
PIK3CG	phosphatidylinositol-4,5-bisphosphate 3-kinase, catalytic subunit gamma	
PIN1	peptidylprolyl cis/trans isomerase, NIMA-interacting 1	
PLA2G1B	phospholipase A2, group IB (pancreas)	Regulation
PLA2G6	phospholipase A2, group VI (cytosolic, calcium-independent)	Cell Functions
PLAU	plasminogen activator, urokinase	Senescence
PLAUR	plasminogen activator, urokinase receptor	
PMCH	pro-melanin-concentrating hormone	Cell Functions
PNMA1	paraneoplastic Ma antigen 1	
POU2AF1	POU class 2 associating factor 1	
POU2F2	POU class 2 homeobox 2	
PPARG	peroxisome proliferator-activated receptor gamma	
PPBP	pro-platelet basic protein (chemokine (C-X-C motif) ligand 7)	Chemokines

PRAME	preferentially expressed antigen in melanoma	
PRF1	perforin 1 (pore forming protein)	Cytotoxicity, Pathogen Defense
PRG2	proteoglycan 2, bone marrow (natural killer cell activator, eosinophil granule major basic protein)	Pathogen Defense
PRKCD	protein kinase C, delta	Senescence
PRKCE	protein kinase C, epsilon	Macrophage Functions
PRM1	protamine 1	
PSEN1	presenilin 1	Cell Functions
PSEN2	presenilin 2 (Alzheimer disease 4)	Macrophage Functions
PSMB10	proteasome (prosome, macropain) subunit, beta type, 10	
PSMB7	proteasome (prosome, macropain) subunit, beta type, 7	Antigen Processing
PSMB8	proteasome (prosome, macropain) subunit, beta type, 8 (large multifunctional peptidase 7)	Chemokines
PSMB9	proteasome (prosome, macropain) subunit, beta type, 9 (large multifunctional peptidase 2)	Antigen Processing
PSMD7	proteasome (prosome, macropain) 26S subunit, non-ATPase, 7	
PTGDR2	prostaglandin D2 receptor 2	Cell Functions
PTGS2	prostaglandin-endoperoxide synthase 2 (prostaglandin G/H synthase and cyclooxygenase)	Cytokines
PTPRC	protein tyrosine phosphatase, receptor type, C	B-Cell Functions, T-Cell Functions
PVR	poliovirus receptor	Regulation
PYCARD	PYD and CARD domain containing	
RAG1	recombination activating gene 1	B-Cell Functions, T-Cell Functions
REL	v-rel reticuloendotheliosis viral oncogene homolog (avian)	Regulation
RELA	v-rel reticuloendotheliosis viral oncogene homolog A (avian)	
RELB	v-rel reticuloendotheliosis viral oncogene homolog B	Regulation

REPS1	RALBP1 associated Eps domain containing 1	Cell Functions
RIPK2	receptor-interacting serine-threonine kinase 2	
ROPN1	rhophilin associated tail protein 1	
RORA	RAR-related orphan receptor A	Regulation
RORC	RAR-related orphan receptor C	Cell Functions
RPS6	ribosomal protein S6	Cell Functions
RRAD	Ras-related associated with diabetes	Cell Functions
RUNX1	runt-related transcription factor 1	Regulation
RUNX3	runt-related transcription factor 3	Regulation
S100A12	S100 calcium binding protein A12	
S100A7	S100 calcium binding protein A7	
S100A8	S100 calcium binding protein A8	
S100B	S100 calcium binding protein B	
SAA1	serum amyloid A1	
SBNO2	strawberry notch homolog 2 (Drosophila)	Macrophage Functions
SELE	selectin E	Regulation
SELL	selectin L	Regulation
SELPLG	selectin P ligand	
SEMG1	semenogelin I	
SERPINB2	serpin peptidase inhibitor, clade B (ovalbumin), member 2	Senescence
SERPING1	serpin peptidase inhibitor, clade G (C1 inhibitor), member 1	
SH2B2	SH2B adaptor protein 2	Regulation
SH2D1A	SH2 domain containing 1A	
SH2D1B	SH2 domain containing 1B	Leukocyte Functions
SIGIRR	single immunoglobulin and toll-interleukin 1 receptor (TIR) domain	
SIGLEC1	sialic acid binding Ig-like lectin 1, sialoadhesin	Transporter Functions

SLAMF1	signaling lymphocytic activation molecule family member 1	
SLAMF6	SLAM family member 6	
SLAMF7	SLAM family member 7	
SLC11A1	solute carrier family 11 (proton-coupled divalent metal ion transporters), member 1	Macrophage Functions
SMAD2	SMAD family member 2	Cell Functions
SMAD3	SMAD family member 3	Regulation
SMPD3	sphingomyelin phosphodiesterase 3, neutral membrane (neutral sphingomyelinase II)	Cell Functions
SOCS1	suppressor of cytokine signaling 1	B-Cell Functions, Cell Functions, T-Cell Functions
SPA17	sperm autoantigenic protein 17	
SPACA3	sperm acrosome associated 3	
SPANXB1	SPANX family, member B1	
SPINK5	serine peptidase inhibitor, Kazal type 5	Regulation
SPN	sialophorin	Regulation
SPO11	SPO11 meiotic protein covalently bound to DSB homolog (S. cerevisiae)	
SPP1	secreted phosphoprotein 1	Cytokines
SSX1	synovial sarcoma, X breakpoint 1	
SSX4	synovial sarcoma, X breakpoint 4	
ST6GAL1	ST6 beta-galactosamide alpha-2,6-sialyltranferase 1	
STAT1	signal transducer and activator of transcription 1, 91kDa	Chemokines, Regulation
STAT2	signal transducer and activator of transcription 2, 113kDa	Chemokines, Regulation
STAT3	signal transducer and activator of transcription 3 (acute-phase response factor)	Chemokines, Regulation
STAT4	signal transducer and activator of transcription 4	Chemokines, Regulation, T-Cell Functions
STAT5B	signal transducer and activator of transcription 5B	Chemokines, Regulation

STAT6	signal transducer and activator of transcription 6, interleukin-4 induced	Chemokines, Regulation, T-Cell Functions
SYCP1	synaptonemal complex protein 1	
SYK	spleen tyrosine kinase	Macrophage Functions
SYT17	synaptotagmin XVII	Cell Functions
TAB1	TGF-beta activated kinase 1/MAP3K7 binding protein 1	
TAL1	T-cell acute lymphocytic leukemia 1	Regulation
TANK	TRAF family member-associated NFKB activator	
TAP1	transporter 1, ATP-binding cassette, sub-family B (MDR/TAP)	Antigen Processing
TAP2	transporter 2, ATP-binding cassette, sub-family B (MDR/TAP)	Antigen Processing
TAPBP	TAP binding protein (tapasin)	Antigen Processing
TARP	TCR gamma alternate reading frame protein	Cell Functions
TBK1	TANK-binding kinase 1	
TBX21	T-box 21	Regulation, T-Cell Functions
TCF7	transcription factor 7 (T-cell specific, HMG-box)	Regulation
TFE3	transcription factor binding to IGHM enhancer 3	
TFEB	transcription factor EB	
TFRC	transferrin receptor (p90, CD71)	
TGFB1	transforming growth factor, beta 1	Interleukins, Regulation
TGFB2	transforming growth factor, beta 2	Interleukins
THBD	thrombomodulin	Leukocyte Functions
THBS1	thrombospondin 1	Antigen Processing, Cell Cycle, Regulation
THY1	Thy-1 cell surface antigen	
TICAM1	toll-like receptor adaptor molecule 1	Macrophage Functions
TICAM2	toll-like receptor adaptor molecule 2	

TIGIT	T cell immunoreceptor with Ig and ITIM domains	T-Cell Functions
TIRAP	toll-interleukin 1 receptor (TIR) domain containing adaptor protein	
TLR1	toll-like receptor 1	Microglial Functions, TLR
TLR10	toll-like receptor 10	TLR
TLR2	toll-like receptor 2	TLR
TLR3	toll-like receptor 3	Microglial Functions, TLR
TLR4	toll-like receptor 4	Microglial Functions, TLR
TLR5	toll-like receptor 5	TLR
TLR6	toll-like receptor 6	TLR
TLR7	toll-like receptor 7	Microglial Functions, TLR
TLR8	toll-like receptor 8	TLR
TLR9	toll-like receptor 9	TLR
TMEFF2	transmembrane protein with EGF-like and two follistatin-like domains 2	
TNF	tumor necrosis factor	Interleukins, TNF Superfamily
TNFAIP3	tumor necrosis factor, alpha-induced protein 3	TNF Superfamily
TNFRSF10B	tumor necrosis factor receptor superfamily, member 10b	TNF Superfamily
TNFRSF10C	tumor necrosis factor receptor superfamily, member 10c, decoy without an intracellular domain	TNF Superfamily
TNFRSF11A	tumor necrosis factor receptor superfamily, member 11a, NFKB activator	TNF Superfamily
TNFRSF11B	tumor necrosis factor receptor superfamily, member 11b	TNF Superfamily
TNFRSF12A	tumor necrosis factor receptor superfamily, member 12A	TNF Superfamily
TNFRSF13B	tumor necrosis factor receptor superfamily, member 13B	Chemokines, TNF Superfamily
TNFRSF13C	tumor necrosis factor receptor superfamily, member 13C	Regulation, TNF Superfamily
TNFRSF14	tumor necrosis factor receptor superfamily, member 14	B-Cell Functions, Regulation, T-Cell Functions, TNF Superfamily

TNFRSF17	tumor necrosis factor receptor superfamily, member 17	Cell Functions, TNF Superfamily
TNFRSF18	tumor necrosis factor receptor superfamily, member 18	TNF Superfamily
TNFRSF1A	tumor necrosis factor receptor superfamily, member 1A	Chemokines, TNF Superfamily
TNFRSF1B	tumor necrosis factor receptor superfamily, member 1B	Chemokines, TNF Superfamily
TNFRSF4	tumor necrosis factor receptor superfamily, member 4	TNF Superfamily
TNFRSF8	tumor necrosis factor receptor superfamily, member 8	TNF Superfamily
TNFRSF9	tumor necrosis factor receptor superfamily, member 9	TNF Superfamily
TNFSF10	tumor necrosis factor (ligand) superfamily, member 10	Cell Cycle, Cytokines, Regulation, TNF Superfamily
TNFSF11	tumor necrosis factor (ligand) superfamily, member 11	TNF Superfamily, Transporter Functions
TNFSF12	tumor necrosis factor (ligand) superfamily, member 12	Chemokines, TNF Superfamily
TNFSF13	tumor necrosis factor (ligand) superfamily, member 13	Regulation, TNF Superfamily
TNFSF13B	tumor necrosis factor (ligand) superfamily, member 13b	Regulation, TNF Superfamily
TNFSF14	tumor necrosis factor (ligand) superfamily, member 14	Cytokines, Regulation, T-Cell Functions, TNF Superfamily
TNFSF15	tumor necrosis factor (ligand) superfamily, member 15	Chemokines, TNF Superfamily
TNFSF18	tumor necrosis factor (ligand) superfamily, member 18	B-Cell Functions, Cell Functions, T-Cell Functions, TNF Superfamily
TNFSF4	tumor necrosis factor (ligand) superfamily, member 4	Chemokines, TNF Superfamily
TNFSF8	tumor necrosis factor (ligand) superfamily, member 8	Cytokines, TNF Superfamily
TOLLIP	toll interacting protein	
TP53	tumor protein p53	T-Cell Functions
TPSAB1	tryptase alpha/beta 1	Cell Functions
TPTE	transmembrane phosphatase with tensin homology	
TRAF2	TNF receptor-associated factor 2	
TRAF3	TNF receptor-associated factor 3	
TRAF6	TNF receptor-associated factor 6, E3 ubiquitin protein ligase	

TREM1	triggering receptor expressed on myeloid cells 1	
TREM2	triggering receptor expressed on myeloid cells 2	
TTK	TTK protein kinase	
TXK	TXK tyrosine kinase	
TXNIP	thioredoxin interacting protein	
TYK2	tyrosine kinase 2	Cytokines, Pathogen Defense, Regulation
UBC	ubiquitin C	
ULBP2	UL16 binding protein 2	Regulation
USP9Y	ubiquitin specific peptidase 9, Y-linked	Cell Functions
VCAM1	vascular cell adhesion molecule 1	Adhesion, Regulation
VEGFA	vascular endothelial growth factor A	Cytokines, Leukocyte Functions
VEGFC	vascular endothelial growth factor C	
XCL2	chemokine (C motif) ligand 2	Chemokines
XCR1	chemokine (C motif) receptor 1	Chemokines
YTHDF2	YTH domain family, member 2	
ZAP70	zeta-chain (TCR) associated protein kinase 70kDa	
ZNF205	zinc finger protein 205	Cell Functions
**Internal Reference Genes**		
ABCF1	ATP-binding cassette, sub-family F (GCN20), member 1	
AGK	acylglycerol kinase	
ALAS1	aminolevulinate, delta-, synthase 1	
AMMECR1L	AMME chromosomal region gene 1-like	
CC2D1B	coiled-coil and C2 domain containing 1B	
CNOT10	CCR4-NOT transcription complex, subunit 10	
CNOT4	CCR4-NOT transcription complex, subunit 4	
COG7	component of oligomeric golgi complex 7	

DDX50	DEAD (Asp-Glu-Ala-Asp) box polypeptide 50
DHX16	DEAH (Asp-Glu-Ala-His) box polypeptide 16
DNAJC14	DnaJ (Hsp40) homolog, subfamily C, member 14
EDC3	enhancer of mRNA decapping 3 homolog (S. cerevisiae)
EIF2B4	eukaryotic translation initiation factor 2B, subunit 4 delta, 67kDa
ERCC3	excision repair cross-complementing rodent repair deficiency, complementation group 3
FCF1	FCF1 small subunit (SSU) processome component homolog (S. cerevisiae)
G6PD	glucose-6-phosphate dehydrogenase
GPATCH3	G patch domain containing 3
GUSB	glucuronidase, beta
HDAC3	histone deacetylase 3
HPRT1	hypoxanthine phosphoribosyltransferase 1
MRPS5	mitochondrial ribosomal protein S5
MTMR14	myotubularin related protein 14
NOL7	nucleolar protein 7, 27kDa
NUBP1	nucleotide binding protein 1
POLR2A	polymerase (RNA) II (DNA directed) polypeptide A, 220kDa
PPIA	peptidylprolyl isomerase A (cyclophilin A)
PRPF38A	PRP38 pre-mRNA processing factor 38 (yeast) domain containing A
SAP130	Sin3A-associated protein, 130kDa
SDHA	succinate dehydrogenase complex, subunit A, flavoprotein (Fp)
SF3A3	splicing factor 3a, subunit 3, 60kDa
TBP	TATA box binding protein
TLK2	tousled-like kinase 2
TMUB2	transmembrane and ubiquitin-like domain containing 2
TRIM39	tripartite motif containing 39

TUBB	tubulin, beta class I
USP39	ubiquitin specific peptidase 39
ZC3H14	zinc finger CCCH-type containing 14
ZKSCAN5	zinc finger with KRAB and SCAN domains 5
ZNF143	zinc finger protein 143
ZNF346	zinc finger protein 346

Table S3. Functional categories and related numbers of genes profiled using the NanoString PanCancer Immune panel.

**Categories**	**Number of Genes**
Adhesion	25
Antigen Processing	22
B-Cell Functions	25
Cell Cycle	13
Cell Functions	71
Chemokines	99
Complement	15
Cytokines	56
Cytotoxicity	10
Interleukins	38
Leukocyte Functions	8
Macrophage Functions	15
Microglial Functions	5
NK Cell Functions	31
Pathogen Defense	12
Regulation	155
Senescence	12
T-Cell Functions	70
TLR	11
TNF Superfamily	30
Transporter Functions	22

Table S4. Primary annotations and related number of genes profiled using the NanoString PanCancer Immune panel.

Primary Annotations	Number of Genes
Acute-phase response	8
Adaptive immune response	109
Adhesion	25
Antigen processing and presentation	21
Anti-inflammatory cytokines	10
Autophagic vacuole formation	1
Autophagy induction by intracellular pathogens	1
Basic cell functions	61
B-cell activation	8
B-cell differentiation	5
B-cell proliferation	1
B-cell receptor signaling pathway	3
CD molecules	238
CD8-positive	2
Cell cycle arrest	1
Cell cycle checkpoint and cell cycle arrest	2
Cell Type specific	109
Chemokines and receptors	88
Chronic inflammatory response	6
Chronic inflammatory response to antigenic stimulus	1
Complement pathway	15
Co-Regulators of autophagy and apoptosis/cell cycle	6
Cytokines and receptors	62
Cytotoxicity	10
Defense response to bacterium	2
Defense response to fungus	2
Defense response to tumor cell	1
Defense response to virus	10
DNA damage checkpoint	1
G1/S transition of mitotic cell cycle	1
G2 phase and G2/M transition	1
Genes linking autophagosome to lysosome	1
Genes responsible for protein transport	3
Humoral immune response	41
Immune response to tumor cell	1
Immunosuppression	3

Induction of apoptosis	1
Inflammatory response	29
Inflammatory response to antigenic stimulus	5
Innate immune response	200
Interleukins	42
Leukocyte activation	2
Leukocyte migration	5
Lipid transporter activity	1
M phase of mitotic cell cycle	1
Macrophage activation	6
Microglial cell activation	5
Negative regulation of antigen processing	1
Negative regulation of cell cycle	1
Negative regulation of immune response	9
NK cell activation	2
NK cell functions	15
Phagocytosis	3
Phagocytosis recognition and engulfment	4
Phagosome processing	1
Phagyocytosis signal transduction	2
Positive regulation of B-cell proliferation	1
Positive regulation of immune response	10
Positive regulation of macrophages	1
Protein ubiquitination	1
Receptors involved in phagocytosis	7
Regulation of cell cycle	3
Regulation of immune response	63
Regulation of inflammatory response	25
Regulators of T-cell activation	9
Regulators of Th1 and Th2 development	6
Response to drug	4
S phase and DNA replication	1
Senescence initiators	3
Senescence initiators interferon related	4
Senescence pathway	6
T-cell activation	12
T-cell anergy	2
T-cell differentiation	15
T-cell mediated immunity	1
T-cell polarization	12
T-cell proliferation	11

T-cell receptor signaling pathway	1
T-cell regulators	4
Th1 & Th2 differentiation	6
Th1 orientation	16
Th2 orientation	9
TNF superfamily members and their receptors	30
Toll-like receptor	11
Transcription factors	20
Transcriptional regulators	15

Table S5. Immune subtype and related number of genes profiled using the NanoString PanCancer Immune panel.

Immune subtype	Number of Genes
Adaptive immune response	104
Inflammation	66
Humoral immune response	41
Innate immune response	200

Table S6. Overall distribution of T-cell density in tumour and non-tumour background tissue across HIV+ (n=63) and HIV- patients (n=66).

**Multiplex IHC (cells/mm²)** Median (range)		**HIV-**	**HIV+**	**p value**
**Tumour**	$CD4^{+}FoxP3^{-}$	93 (0-2484)	117 (0-392)	0.962
	$CD4^{+}FoxP3^{+}$	3 (0-26)	25 (0-270)	**<0.001***
	$CD8^{+}PD\text{-}1^{-}$	26 (0-900)	94 (0-800)	**0.002***
	$CD8^{+}PD\text{-}1^{+}$	7 (0-35)	31 (0-180)	**<0.001***
**Multiplex IHC (cells/mm²)** Median (range)		**HIV-**	**HIV+**	**p value**
**Non tumour**	$CD4^{+}FoxP3^{-}$	276 (2-2380)	163 (8-700)	**0.037***
	$CD4^{+}FoxP3^{+}$	3 (0-55)	8 (0-90)	0.104
	$CD8^{+}PD\text{-}1^{-}$	91 (3-1240)	229 (8-784)	**<0.001***
	$CD8^{+}PD\text{-}1^{+}$	2 (0-780)	60 (0-204)	**<0.001***

Table S7. Overall distribution of T-cell density in tumour and non-tumour background tissue across Child-Turcotte-Pugh (CTP) A (n=79) and CTP B-C patients (n=48).

**Multiplex IHC (cells/mm²)** Median (range)		**Child Pugh A**	**Child Pugh B-C**	**p value**
**Tumour**	$CD4^{+}FoxP3^{-}$	134 (0-392)	89 (0-320)	0.455
	$CD4^{+}FoxP3^{+}$	33 (0-270)	16 (0-140)	0.130
	$CD8^{+}PD\text{-}1^{-}$	98 (0-650)	50 (5-800)	0.267
	$CD8^{+}PD\text{-}1^{+}$	33 (0-180)	30 (2-98)	0.290
**Multiplex IHC (cells/mm²)** Median (range)		**Child Pugh A**	**Child Pugh B-C**	**p value**
**Non tumour**	$CD4^{+}FoxP3^{-}$	400 (163-1609)	40 (24-1600)	0.941
	$CD4^{+}FoxP3^{+}$	40 (0-253)	25 (8-200)	0.385
	$CD8^{+}PD\text{-}1^{-}$	514 (89-2246)	588 (100-2500)	0.819
	$CD8^{+}PD\text{-}1^{+}$	98 (8-270)	100 (30-172)	0.628

Table S8. Characteristics of patients who underwent transcriptomics and TCR sequencing analysis.

Baseline characteristics	Nanostring Dataset n (%)		ImmunoSeq Dataset n (%)	
	HIV + (n=23)	HIV – (n=25)	HIV + (n=16)	HIV – (n=16)
Age at diagnosis, median (interquartile range)	52 (41-64)	58 (44-71)	53 (42-64)	58 (44-71)
Gender (male)	19 (83)	20 (80)	16 (100)	15 (97)
Cirrhosis	20 (87)	21 (83)	16 (100)	16 (100)
Etiology (viral)	22 (95)	19 (77)	15 (97)	13 (81)
Child-Pugh class				
A	7 (30)	6 (29)	10 (62)	9 (56)
B	5 (21)	5 (25)	4 (25)	7 (43)
C	0	1 (4)	0	0
Barcelona Clinic Liver Cancer stage				
A	5 (21)	5 (25)	7 (43)	6 (37)
B	1 (4)	1 (4)	0	3 (18)
C	1 (4)	0	3 (18)	2 (12)
D	0	1 (4)	0	0
Portal vein thrombosis	0 (0)	0 (0)	1 (6)	1 (6)
Extrahepatic spread	1 (4)	0	3 (18)	1 (6)
Alfa-fetoprotein (>400 IU/mL)	2 (8)	0	1 (6)	3 (18)
