# Supplementary material for: Integrated phenotyping of the anti-cancer immune response in HIV-associated hepatocellular carcinoma
Source: JHEP Rep. 2023 Mar 22;5(7):100741. doi: 10.1016/j.jhepr.2023.100741 (PMC10238838; doi:10.1016/j.jhepr.2023.100741)
Supplement: Multimedia component 2 [file mmc2.docx]

**JHEP Reports**

**CTAT methods**

Tables for a “Complete, Transparent, Accurate and Timely account” (CTAT) are now mandatory for all revised submissions. The aim is to enhance the reproducibility of methods.

- Only include the parts relevant to your study
- Refer to the CTAT in the main text as ‘Supplementary CTAT Table’
- Do not add subheadings
- Add as many rows as needed to include all information
- Only include one item per row

**If the CTAT form is not relevant to your study, please outline the reasons why:**

|  |
| --- |

- 1. **Antibodies**

| **Name** | **Citation** | **Supplier** | **Cat no.** | **Clone no.** |
| --- | --- | --- | --- | --- |
| **PD-L1** | Ref 23  Pinato DJ et al. Annal Oncol. 2018;29:1486-8. | Cell Signalling, MA, USA | 13684 | E1L3N |
| **CD4** | Ref 23  Pinato DJ et al. Annal Oncol. 2018;29:1486-8. | Spring Biosciences, Pleasanton, CA, USA |  | SP35 |
| **CD8** | Ref 23  Pinato DJ et al. Annal Oncol. 2018;29:1486-8. | Spring Biosciences, Pleasanton, CA, USA |  | SP239 |
| **FOXP3** | Ref 23  Pinato DJ et al. Annal Oncol. 2018;29:1486-8. | Biolegend, San Diego, California, USA |  | 259D |
| **PD-1** | Ref 23  Pinato DJ et al. Annal Oncol. 2018;29:1486-8. | Spring Biosciences, Pleasanton, CA, USA |  | NAT 105/E3 |

- 1. **Cell lines**

| **Name** | **Citation** | **Supplier** | **Cat no.** | **Passage no.** | **Authentication test method** |
| --- | --- | --- | --- | --- | --- |
| **N/A** |  |  |  |  |  |

- 1. **Organisms**

| **Name** | **Citation** | **Supplier** | **Strain** | **Sex** | **Age** | **Overall n number** |
| --- | --- | --- | --- | --- | --- | --- |
| **N/A** |  |  |  |  |  |  |

- 1. **Sequence based reagents**

| **Name** | **Sequence** | **Supplier** |
| --- | --- | --- |
| **N/A** |  |  |

- 1. **Biological samples**

| **Description** | **Source** | **Identifier** |
| --- | --- | --- |
| Formalin-fixed paraffin-embedded (FFPE) material from diagnostic biopsy (n=20) or surgical specimens (n=109) | Multi-centre biorepository as detailed in Supplementary Table 1 | Anonymised samples |

- 1. **Deposited data**

| **Name of repository** | **Identifier** | **Lin** |
| --- | --- | --- |
| **N/A** |  |  |

- 1. **Software**

| **Software name** | **Manufacturer** | **Version** |
| --- | --- | --- |
| **SPSS** | IBM Inc., Chicago, IL, USA | 26.0 |
| **GraphPad Prism** | GraphPad software Inc., La Jolla, CA, USA | 9.0 |

- 1. **Other (*e.g*. drugs, proteins, vectors etc.)**

| **N/A** |  |  |
| --- | --- | --- |
|  |  |  |

- 1. **Please provide the details of the corresponding methods author for the manuscript:**

| Dr David J. Pinato, MD MRes MRCP PhD  Clinical Senior Lecturer and Consultant in Medical Oncology  Imperial College London Hammersmith Campus,  Du Cane Road, W12 0HS, London (UK)  Tel: +44 020 83833720 E-mail: david.pinato@imperial.ac.uk |
| --- |

**2.0 Please confirm for randomised controlled trials all versions of the clinical protocol are included in the submission. These will be published online as supplementary information.**

| **N/A** |
| --- |
